# Synthesis of Highly Fluorinated Arene Complexes of [Rh(Chelating Phosphine)]^+^ Cations, and their use in Synthesis and Catalysis

**DOI:** 10.1002/chem.201904668

**Published:** 2020-02-11

**Authors:** Alasdair I. McKay, James Barwick‐Silk, Max Savage, Michael C. Willis, Andrew S. Weller

**Affiliations:** ^1^ Department of Chemistry Chemistry Research Laboratory University of Oxford Mansfield Road Oxford OX1 3TA UK; ^2^ Department of Chemistry University of York York YO10 5DD UK

**Keywords:** catalysis, fluoroarene, phosphine, rhodium, x-ray

## Abstract

The synthesis of rhodium complexes with weakly binding highly fluorinated benzene ligands is described: 1,2,3‐F_3_C_6_H_3_, 1,2,3,4‐F_4_C_6_H_2_ and 1,2,3,4,5‐F_5_C_6_H are shown to bind with cationic [Rh(Cy_2_P(CH_2_)_*x*_PCy_2_)]^+^ fragments (*x=*1, 2). Their structures and reactivity with alkenes, and use in catalysis for promoting the Tishchenko reaction of a simple aldehyde, are demonstrated. Key to the synthesis of these complexes is the highly concentrated reaction conditions and use of the [Al{OC(CF_3_)_3_}_4_]^−^ anion.

## Graphical abstract

**Weakly binding highly fluorinated** benzene ligands 1,2,3‐F_3_C_6_H_3_, 1,2,3,4‐F_4_C_6_H_2_ and 1,2,3,4,5‐F_5_C_6_H are shown to bind with cationic [Rh(Cy_2_P(CH_2_)_*x*_PCy_2_)]^+^ fragments (*x=*1, 2), and the resulting complexes have been used in synthesis and catalysis.

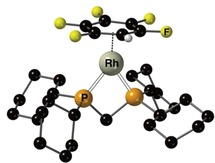

## Introduction

**Scheme 1:**
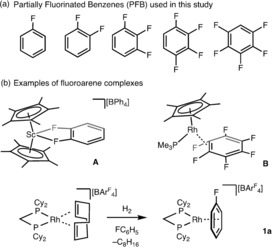
(a) Partially fluorinated benzenes (PFBs); (b) examples of PFB complexes.

Partially fluorinated benzenes (PFBs), exemplified by fluorobenzene (FC_6_H_5_) through to pentafluorobenzene (F_5_C_6_H) (Scheme 1 a), have emerged as versatile solvents in contemporary synthetic chemistry and catalysis. They also act as innocent weakly coordinating ligands with transition metal fragments that offer up operationally unsaturated catalytic systems.^1^ Coordination of the PFB through the fluorine substituents (κ^n^_F_ coordination, for example, **A**, Scheme 1 b) has been reported for early transition metals,^2^ whilst coordination via the arene ring (η^n^ coordination) is principally observed in mid‐ to late‐transition metal complexes.^3^ The majority of structurally characterized metal PFB complexes feature η‐FC_6_H_5_ or η‐1,2‐F_2_C_6_H_4_.1a, 4 While a handful of mid‐ to late‐transition metal hexafluorobenzene (C_6_F_6_) complexes have also been structurally characterized, that adopt η^2^ (e.g., **B**) or η^6^ bonding modes,^5^ the only example of F_5_C_6_H coordinated to a metal center that has been structurally characterized is [(PEt_3_)_2_Ni]_2_(μ‐η^2^:η^2^‐F_5_C_6_H),^6^ with no examples (until recently, vide infra) of F_4_C_6_H_2_ or F_3_C_6_H_3_. Transition metal complexes of PFBs are also of interest with regard to the role such complexes play as intermediates in C−F activation processes of fluoroaromatics.^7^

Gas‐phase mass spectrometric experiments have shown that binding of PFBs with a transition metal fragment through the π‐arene face decreases by roughly 5 kcal mol^−1^ with each added fluorine substituent in Cr^+^‐based systems.^8^ For cationic rhodium diphosphines based upon [Rh(*i*Bu_2_P(CH_2_)_*x*_P*i*Bu_2_)(F_n_C_6_H_6−*n*_)][BAr^F^_4_] [Ar^F^=3,5‐C_6_H_3_(CF_3_)_2_] the relative binding strength also decreases with each added fluorine substituent, as measured by solution equilibrium and gas‐phase collision induced dissociation experiments, using electrospray ionization mass spectrometry (ESI‐MS).^9^ This weak binding with increased fluorine‐substitution means that π‐complexes of 1,2,3,4‐F_4_C_6_H_2_ and F_5_C_6_H were found to be inaccessible in solution, due to competition for π‐complexation by the [BAr^F^_4_]^−^ anion, that gives zwitterionic complexes such as [Rh(*i*Bu_2_PCH_2_CH_2_P*i*Bu_2_){η^6^‐(3,5‐(CF_3_)_2_C_6_H_3_)BAr^F^_3_}].^10^ This, and other,1b studies also reported that the binding strength of η‐FC_6_H_5_ increased with reduction of the diphosphine bite angle.^9^ Thus small bite angle diphosphines, such as Cy_2_PCH_2_PCy_2_, promote the strongest binding of fluoroarenes with {Rh(L_2_)}^+^ fragments.

**Scheme 2:**
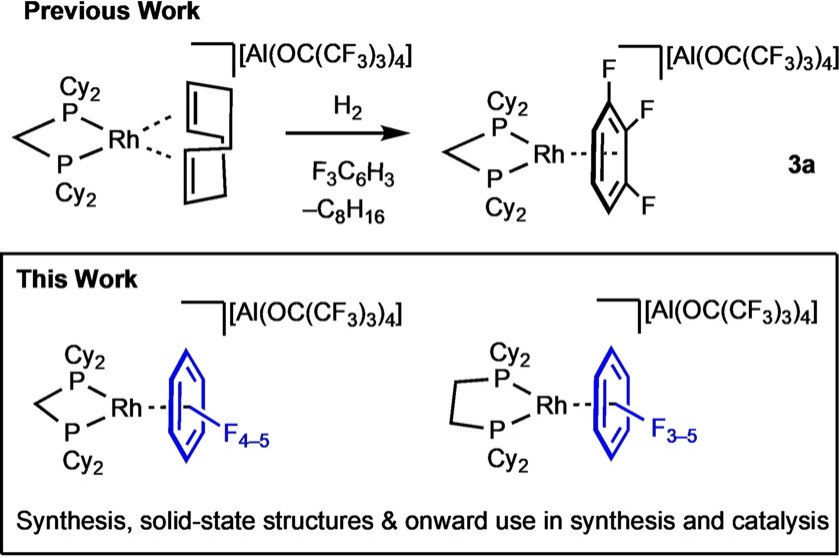
A previously reported complex of F_3_C_6_H_3_ ligation, and the new complexes reported in this work.

We have reported the synthesis of fluorobenzene (FC_6_H_5_) and difluorobenzene (1,2‐F_2_C_6_H_4_) complexes [Rh(L_2_)(F_n_C_6_H_6−*n*_)][BAr^F^_4_] (L_2_=monodentate or bidentate phosphine) as drop–in precatalysts for challenging catalytic transformations.1b, 3, 11 These are accessed via hydrogenation of a NBD or COD (norbornadiene/cyclooctadiene) precursor in the arene of choice (e.g., **1 a**, Scheme 1 b). Others have reported related fluoroarene complexes.5f, 6, 12 While the fluoroarene in these complexes can be replaced by relatively stronger ligands (e.g. acetone) there are instances when substitution does not take place as the arene binds too strongly,11e especially with smaller bite‐angle diphosphine ligands. To overcome this we have recently reported the first structurally characterized metal complex with a weakly binding 1,2,3‐F_3_C_6_H_3_ ligand, [Rh(Cy_2_PCH_2_PCy_2_)(1,2,3‐F_3_C_6_H_3_)][Al{OC(CF_3_)_3_}_4_] **3 a** (Scheme 2).^13^ Displacement of the fluoroarene by H_3_B**⋅**NMe_3_ generates the corresponding sigma amine‐borane complex. The use of the non‐coordinating [Al{OC(CF_3_)_3_}_4_]^−^ anion, popularized by Krossing,^14^ is crucial to this reactivity, as the more commonly‐used [BAr^F^_4_]^−^ anion binds competitively with both 1,2,3‐F_3_C_6_H_3_ and the amine‐borane H_3_B⋅NMe_3_, thus quenching reactivity.

We now report an extension to this synthetic methodology to prepare and structurally characterize fluoroarene complexes of 1,2,3‐F_3_C_6_H_3_, 1,2,3,4‐F_4_C_6_H_2_ and F_5_C_6_H with two different [Rh(Cy_2_P(CH_2_)_*x*_PCy_2_)]^+^ fragments (*x=*1 and 2), as partnered with the [Al{OC(CF_3_)_3_}_4_]^−^ anion. The weakly binding properties of these arenes is exploited in the synthesis of an alkene complex only previously accessible using molecular solid‐state solid/gas reactivity in the absence of solvent;^15^ and in catalysis for the intermolecular Tishchenko reaction of a simple aldehyde.

## Results and Discussion

### Synthesis and characterization of cationic Rh partially fluorinated benzene complexes

**Scheme 3:**
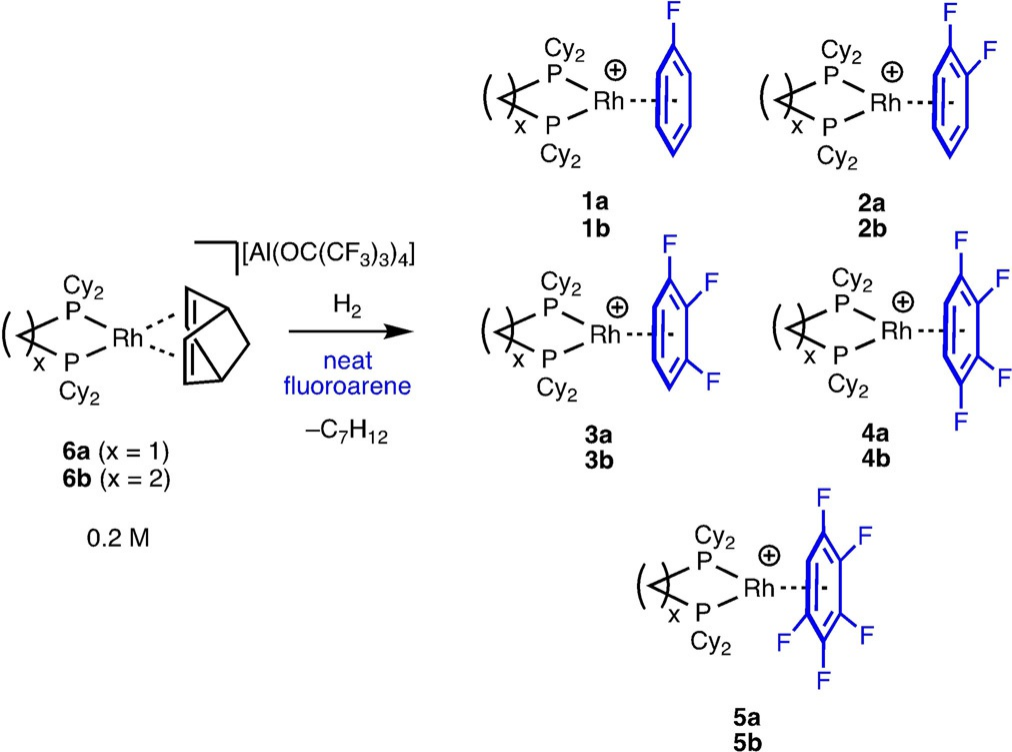
Synthesis of η‐PFB complexes in this work. [Al{OC(CF_3_)_3_}_4_]^−^ anions omitted. Complex **3 a** has been previously reported.^13^

The norbornadiene precursors [Rh(Cy_2_P(CH_2_)_*x*_PCy_2_)(NBD)][Al{OC(CF_3_)_3_}_4_] (*x=*1, **6 a**; *x=*2, **6 b**; NBD=norbornadiene) are conveniently prepared from addition of the corresponding diphosphine to [Rh(NBD)_2_][Al{OC(CF_3_)_3_}_4_]. Addition of H_2_ to these precursors in the appropriate neat partially fluorinated benzene (PFB) reagent afforded the respective PFB complexes [Rh(Cy_2_P(CH_2_)_*x*_PCy_2_)(η‐F_n_C_6_H_6−*n*_)][Al{OC(CF_3_)_3_}_4_] (*n*=1, **1**; *n*=2, **2**; *n*=3, **3**; *n*=4, **4**; *n*=5, **5**), Scheme 3.^16^ Complexes **1 a/b** and **2 a/b** have previously been reported as the [BAr^F^_4_]^−^ analogues.11f, 13, 17 The new complexes were initially characterized by ^31^P{^1^H} NMR spectroscopy of the crude reaction mixture which showed characteristic doublet signals arising from coupling with ^103^Rh. However, the presence of other arene complexes was also revealed in these spectra, i.e., [Rh(Cy_2_P(CH_2_)_*x*_PCy_2_)(arene)][Al{OC(CF_3_)_3_}_4_], which arise from the presence of more strongly coordinating trace arene impurities. We have previously reported GC‐MS analysis of commercially sourced 1,2‐difluorobenzene (1,2‐F_2_C_6_H_4_) which revealed the presence of trace quantities of FClC_6_H_4_ and F(OH)C_6_H_4_.^18^ Whilst additional organometallic products were found to be near negligible in the cases of relatively more strongly ligated complexes **1** and **2** (<5 %), for complexes **3**–**5** considerable quantities are observed (ca. 75 % in the case of **5**).^19^ We previously showed that complex **3 a** can be prepared in high (>95 %) spectroscopic yield and isolated purity if the synthesis is conducted at very high concentrations (ca. 0.17 m, 100 mg in 0.4 cm^3^). This simply decreases the ratio of [impurities]:[Rh],^13^ allowing for binding of the desired arene to dominate. By employing similarly concentrated conditions (≈0.21 m) **5 a/b**, **4 a/b** and **3 b** were prepared in satisfactory spectroscopic yields (>95 %), from which the complexes could be isolated as yellow to orange powders in moderate yield by the addition of pentane. Careful recrystallization of these solutions with pentane results in X‐ray quality crystals of many of these complexes.

With the exception of **1** and **2**, dissolving these new complexes in CD_2_Cl_2_ at room temperature afforded C‐Cl activated species,^20^ as measured by ESI‐MS. Complex **3 b** was stable enough in CD_2_Cl_2_ to permit characterization by ^1^H, ^31^P{^1^H} and ^19^F{^1^H} NMR spectroscopy at 183 K. By contrast ^1^H and ^19^F{^1^H} NMR spectra of **4 a**, **4 b**, **5 a** and **5 b** obtained under these conditions show significant quantities (>25 %) of free 1,2,3,4‐F_4_C_6_H_2_ and F_5_C_6_H, respectively, consistent with CD_2_Cl_2_ solvent competing for binding to the metal center. NMR spectroscopic characterization of these complexes are thus limited to in situ spectra obtained in the appropriate neat fluoroarene. The formation of the arene complexes is signalled by the appearance of a new doublet in the ^31^P{^1^H} NMR spectra, which is downfield shifted relative to that of the parent NBD complexes (**6**) (Table 1), by ≈10 ppm for Cy_2_PCH_2_PCy_2_ complexes and ≈30 ppm for Cy_2_PCH_2_CH_2_PCy_2_ complexes. The magnitude of the ^31^P–^103^Rh coupling constant does not undergo a large change on variation of the arene (a 1–2 Hz reduction per F). In the ^19^F{^1^H} spectra, new, sharp, sets of PFB resonances are observed, which are typically upfield shifted relative to those of the free arene (Table 1). We assign these new resonances to the η‐bound arenes. These observations indicate exchange of bound and free arene to be slow on the NMR timescale at 298 K. In the ^1^H NMR spectra, the signal(s) for the η‐bound PFB in **1**–**3** lie upfield of the solvent, whilst the resonances for η‐F_5_C_6_H in **5** lie downfield shifted of free F_5_C_6_H. The signals for η‐F_4_C_6_H_2_ in **4** were obscured by the solvent.

**Table 1:** ^31^P{^1^H} and ^19^F{^1^H} NMR chemical shifts, and ^31^P‐^103^Rh coupling constants for NBD (**6**) and partially fluorinated arene (**1**–**5**) complexes.

Complex	*δ* ^31^P{^1^H}	*J*(RhP) (Hz)	*δ* ^19^F{^1^H}^[c]^	Ref.
**6 a** ^[a]^	−22.6	133	–	This work
**6 b** ^[a]^	69.8	154	–	This work
**5 a** ^[b]^	−12.4	164	−149.77, −165.00, −167.76	This work
**4 a** ^[b]^	−11.9	166	−149.48, −164.98	This work
**3 a** ^[b]^	−10.9	167	−146.7, −167.1	13
**2 a** ^[a]^	−10.4	168	−146.3	13
**1 a** ^[a]^	−9.9	170	−123.07	This work
**5 b** ^[b]^	97.8	193	−148.68, −163.87, −166.64	This work
**4 b** ^[b]^	97.9	195	−148.98, −163.91	This work
**3 b** ^[b]^	98.3	198	−146.27, −166.27	This work
**2 b** ^[a]^	98.6	199	−145.62	This work
**1 b** ^[a]^	98.5	201	−122.79	This work

[a] Spectrum acquired in CD_2_Cl_2_ at 298 K. [b] Spectrum acquired in neat PFB at 298 K. [c] ^19^F{^1^H} NMR chemical shift of the [Al{OC(CF_3_)_3_}_4_]^−^ anion are omitted.

**Figure 1:**
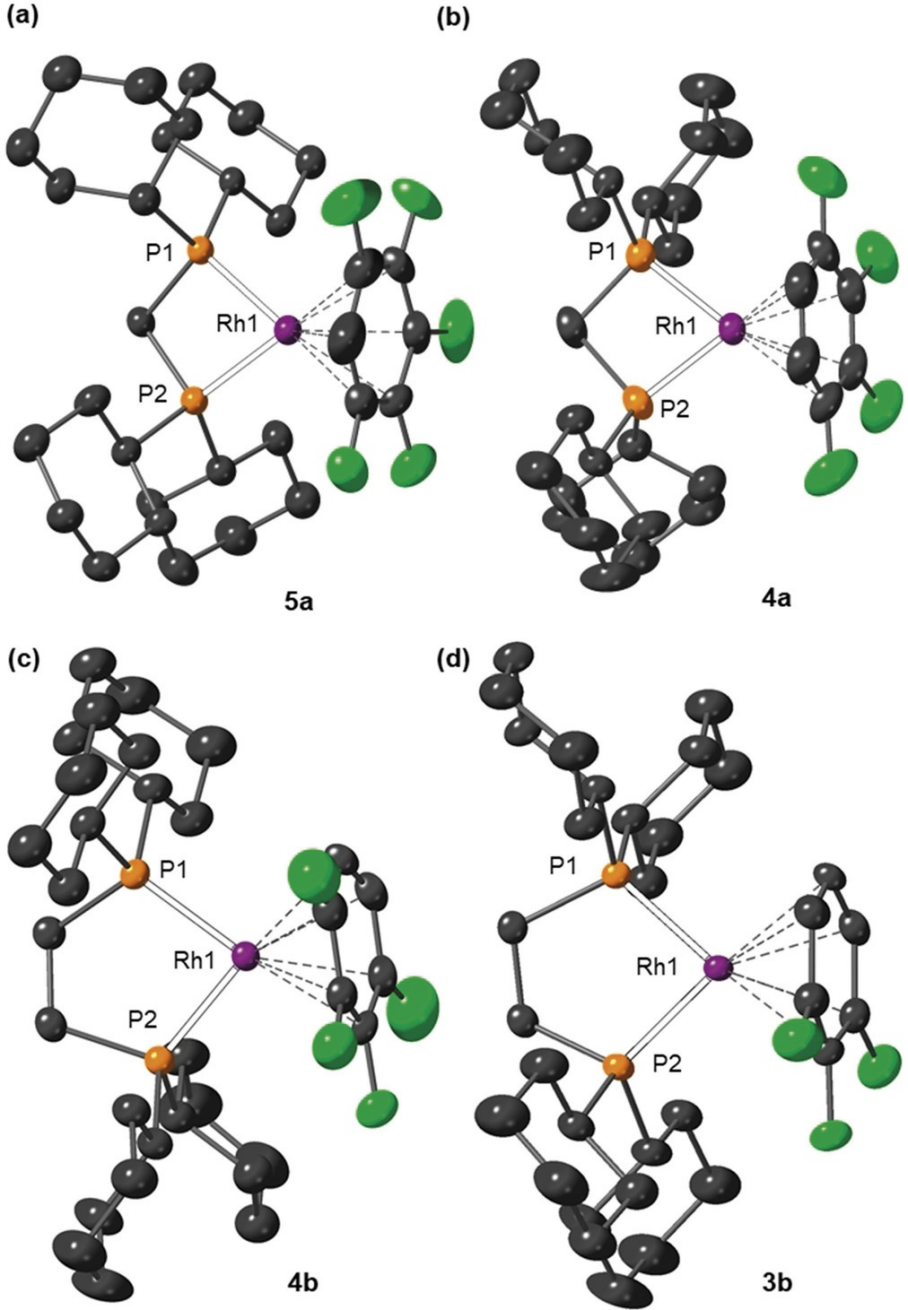
Solid‐state structures of the cationic portion of selected new complexes (50 % displacement ellipsoids). Lower occupancy disorder components (where appropriate) and hydrogen atoms omitted for clarity. Selected bond lengths [Å] and angles [°]: (a) **5 a** Rh‐C_arene_ range: 2.268(7)–2.375(6), Rh1‐P1 2.2514(14), Rh1‐P2 2.2503(14), P1‐Rh1‐P2 72.20(5). (b) **4 a** Rh‐C_arene_ range: 2.258(4)–2.316(6), Rh1‐P1 2.2463(10), Rh1‐P2 2.2416(9), P1‐Rh1‐P2 73.09(4). (c) **4 b** Rh‐C_arene_ range: 2.265(4)–2.345(4), Rh1‐P1 2.2443(10), Rh1‐P2 2.2476(10), P1‐Rh1‐P2 85.08(4). (d) **3 b** Rh‐C_arene_ range: 2.262(3)–2.359(4), Rh1‐P1 2.2407(8), Rh1‐P2 2.2484(9), P1‐Rh1‐P2 84.89(3).

Single crystals of all complexes that were suitable for characterization by X‐ray diffraction were obtained by carefully layering the filtered reaction mixtures with pentane, with the exception of **5 b** which resisted all attempts to recrystallize. Figure 1 shows new complexes **5 a**, **4 a**, **4 b** and **3 b**, and the supporting materials detail **2 b** and **1**. Complexes **4 a** and **4 b** represent the first structurally characterized F_4_C_6_H_2_ metal complexes, whilst there is only a single preceding crystallographically characterized example of an η‐F_5_C_6_H metal complex in the literature: the dinuclear complex, [{(PEt_3_)_2_Ni}_2_(μ‐η^2^:η^2^‐F_5_C_6_H)].^6^ Complex **5 a** crystallizes as a racemic twin in the non‐centrosymmetric space group *P*2_1_2_1_2_1_, and the solid‐state structure of the cation is depicted in Figure 1 a. The F_5_C_6_H ligand is well ordered and the six Rh‐C(arene) distance span the range 2.268(7)–2.375(6) Å. By contrast, complexes **4 a**, **4 b**, **3 b**, **2 b** and **1 b** crystallize in centrosymmetric monoclinic space groups and the fluorobenzene is disordered over several orientations such that it is not appropriate to discuss structural metrics for the arene binding in detail.^21^ The range of Rh−C distances in these structures is 2.258(4)–2.375(6) Å. Nevertheless in all these cases the structural solution is unequivocal and confirm arene binding. The Rh−P bond lengths in complexes **1**–**5** lie in the tight range 2.2316(7)–2.2514(14) Å.

### Relative reactivities of cationic η‐bound PFB Rh complexes.

Having established an effective route to η‐bound PFB complexes of {Rh(Cy_2_PCH_2_CH_2_PCy_2_)}^+^ we sought to demonstrate their synthetic utility for the preparation of new complexes with other weakly‐bound ligands. We have recently reported the synthesis of an isobutene complex [Rh(Cy_2_PCH_2_CH_2_PCy_2_)(η^2^_(C=C)_η^2^_(CH)_‐C_4_H_8_)][BAr^F^_4_] by the solid/gas single‐crystal to single‐crystal reaction of a σ‐alkane complex, [Rh(Cy_2_PCH_2_CH_2_PCy_2_)(η^2^η^2^‐NBA)][BAr^F^_4_] (NBA=norbornane), with isobutene.^15^ The isobutene interacts with the metal center through an alkene and an agostic interaction.^22^ Previous attempts to prepare this material by solution routes were not successful as this alkene will not displace 1,2‐F_2_C_6_H_4_ in the corresponding precursor [Rh(Cy_2_PCH_2_CH_2_PCy_2_)(F_2_C_6_H_4_)][BAr^F^_4_].

**Figure 2:**
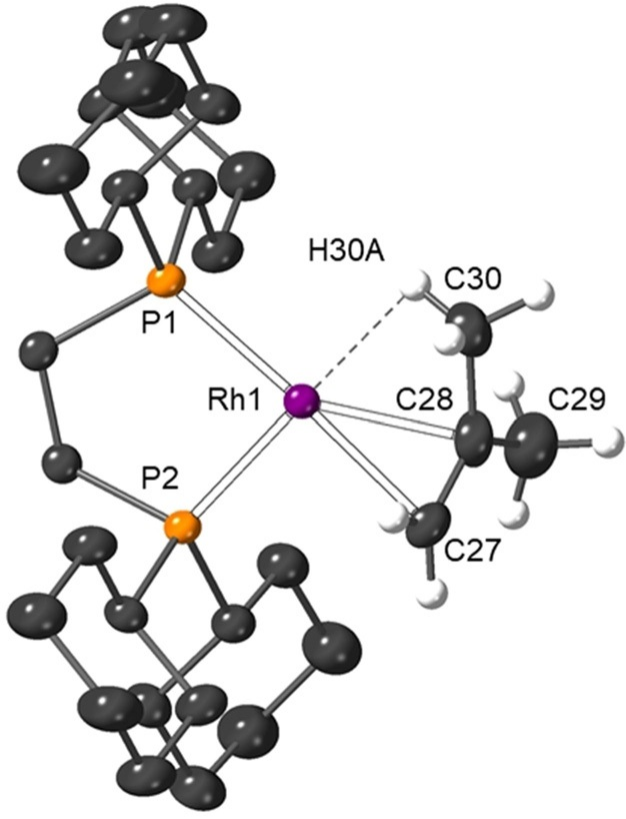
Solid‐state structure of the cationic portion of **7**, hydrogen atoms on the bis‐phosphine ligand omitted. Displacement ellipsoids are shown at the 50 % probability level. Selected bond lengths [Å] and angles [°]: Rh1‐P1 2.2613(6), Rh1‐P2 2.2168(6), Rh1‐C27 2.262(3), Rh1‐C28 2.162(3), Rh1‐C30 2.345(3), Rh1‐H30A 2.012(3), C27‐C28 1.339(5), C28‐C29 1.491(5), C28‐C30 1.503(5), P1‐Rh1‐P2 85.63(2).

Addition of isobutene (1 atm, 298 K) to in situ prepared **5 b** in F_5_C_6_H solution resulted in the appearance of a new resonance in the ^31^P{^1^H} spectrum at *δ* 94.8, *J*(RhP)=181 Hz [cf. [Rh(Cy_2_PCH_2_CH_2_PCy_2_)(η^2^_(C=C)_η^2^_(CH)_‐C_4_H_8_)][BAr^F^_4_] in CD_2_Cl_2_ *δ* 95.3, *J*(RhP)=179 Hz^15^]. Signals for **5 b** were completely absent from the ^1^H, ^31^P{^1^H} and ^19^F{^1^H} NMR spectra. Layering the filtered reaction mixture with pentane afforded yellow‐orange crystals of [Rh(Cy_2_PCH_2_CH_2_PCy_2_)(η^2^_(C=C)_η^2^_(CH)_‐C_4_H_8_)] [Al{OC(CF_3_)_3_}_4_] (**7**), in moderate (50 %) yield, suitable for characterization by single‐crystal X‐ray diffraction. The solid‐state structure is shown in Figure 2 which demonstrates that the cationic portion of **7** is similar to that of [Rh(Cy_2_PCH_2_CH_2_PCy_2_)‐ (η^2^_(C=C)_η^2^_(CH)_‐C_4_H_8_)][BAr^F^_4_], in which the isobutene ligand is disordered over two positions.^15^ However, for **7** the isobutene ligand is not disordered allowing for more reliable structural metrics. The three Rh–C contacts reflect the alkene/agostic interactions [Rh−C27, 2.262(3) Å; Rh−C28, 2.162(3) Å; Rh−C30, 2.345(3) Å], as do the C−C distances [C27−C28 1.339(5); C28−C30 1.503(5) Å]. In CD_2_Cl_2_ solution at 298 K the alkene is undergoing a fluxional process (a 1,3‐shift) that makes all the CH groups equivalent and has been characterized in detail in related propene complexes as being due to an allyl/hydride mechanism via C−H oxidative cleavage of the agostic bond.^23^ At 193 K this is slowed so that broad signals due to C=CH_2_ (*δ* 4.25, 3.32) and agostic (3 H, δ−0.30) are observed. Interestingly while these data are consistent with the structure, they are better resolved than the [BAr^F^_4_]^−^ analogue at 183 K (CD_2_Cl_2_).^15^ While this might suggest that the weakly‐coordinating anion is influencing the relative barriers to the fluxionality, more likely these differences are due to relative solubilities at very low temperatures. Complex **7** slowly decomposed in CD_2_Cl_2_ at room temperature.

**Scheme 4:**
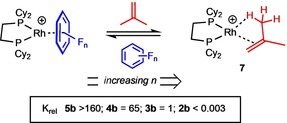
Equilibrium between η‐PFB and η^2^_(C=C)_η^2^_(CH)_‐C_4_H_8_ coordination to {Rh(Cy_2_PCH_2_CH_2_PCy_2_)}^+^ in neat PFB. [Al{OC(CF_3_)_3_}_4_]^−^ anion omitted.

The poor stabilities of complexes **3**–**5** in standard laboratory solvents such as CD_2_Cl_2_ and the presence of trace arene impurities in neat PFB prevented the binding affinities of the PFBs being quantitatively studied by competition reactions between individual pairs of arenes. However, the reactivity of the complexes **2 b**–**5 b** with isobutene presented a strategy to qualitatively study the binding affinities of the series of PFBs by measuring the position of the equilibrium between the η‐PFB complex and complex **7** by ^31^P{^1^H} NMR spectroscopy (Scheme 4). In situ prepared solutions of **2 b–5 b** in the respective PFB solvent were treated with isobutene (1 atm, 298 K) and the ^31^P{^1^H} NMR spectra used to estimate the position of the equilibrium. Sharp signals due to the arene complex and **7** were observed suggesting that the exchange process is slow on the NMR timescale. The data collected demonstrate that the binding affinity of the PFB to the {Rh(Cy_2_PCH_2_CH_2_PCy_2_)}^+^ decreases with increased fluorine substitution on the arene ring. Thus, for F_5_C_6_H (complex **5 b**) only complex **7** is observed on adding isobutene, while for 1,2‐F_2_C_6_H_4_ (complex **2 b**) no isobutene complex was detected.

### Cationic η‐PFB Rh complexes in catalysis

We and others have shown cationic rhodium(I) complexes ligated by small bite‐angle diphosphine and η‐FC_6_H_5_ to be effective pre‐catalysts for intermolecular hydroacylation.11f, 24 However, the substrate scope is limited to aldehydes or alkenes containing a β‐coordinating group.1b, 11f, 11g, 24b, 25 When on the aldehyde, this motif drives the pre‐equilibrium of aldehyde binding by chelation effect and also blocks a vacant site on the corresponding acyl hydride complex that comes from C−H activation. This attenuates catalyst deactivation by an irreversible decarbonylation pathway. As far as we are aware no reactivity has been reported with cationic catalysts and aldehydes lacking a β‐coordinating group, as the pre‐equilibrium disfavors aldehyde coordination especially when the reaction is performed in relatively coordinating solvents such as acetone. Having established the poor binding affinity of η‐F_5_C_6_H to {Rh(Cy_2_PCH_2_PCy_2_)}^+^, i.e., complex **5 a**, it seemed a reasonable assumption that this would encourage the binding of a simple aldehyde such as cyclohexanecarboxaldehyde prior to the C−H activation step.^26^ We probed this C−H activation step by the homocoupling of the aldehyde to form the corresponding ester product—a Tishchenko reaction—which is essentially the hydroacylation of an aldehyde. The reaction of cyclohexanecarboxaldehyde with 5 mol % of in situ formed **5 a** in F_5_C_6_H solution resulted in complete consumption, as measured by NMR spectroscopy, of the aldehyde and formation of the Tishchenko ester product, cyclohexylmethyl cyclohexanecarboxylate, within 5 minutes at 25 °C (Scheme 3). The ester was isolated in 91 % yield after purification by column chromatography, and its characterization data match literature values.^27^ Under analogous conditions, complete consumption of the aldehyde to afford the Tishchenko ester was also observed when the pre‐catalyst was changed to in situ prepared **4 a**, **3 a** or **2 a**. However, the catalysis slows significantly with decreased fluorine substitution of the arene as measured by time to completion for the reaction. These data are consistent with the enhanced binding affinities of the less fluorinated arenes to the cationic Rh fragment as measured by the position of equilibrium with isobutene.

**Table 2:** Tishchenko reaction of cyclohexanecarboxaldehyde catalyzed by [Rh(Cy_2_PCH_2_PCy_2_)(F_*n*_C_6_H_6−*n*_)][Al{OC(CF_3_)_3_}_4_] in PFB.

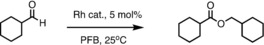			
Pre‐catalyst	Solvent	Time^[a]^	TOF [min^−1^]
**5 a**	F_5_C_6_H	<5	>0.4
**4 a**	F_4_C_6_H_2_	<5	>0.4
**3 a**	F_3_C_6_H_3_	80	0.03
**2 a**	F_2_C_6_H_4_	600	0.003

[a] Time until full consumption of cyclohexanecarboxaldehyde as indicated by periodic monitoring by ^1^H NMR spectroscopy, the ester was the only product observed by ^1^H NMR spectroscopy for all reactions.

## Conclusions

The synthesis of highly fluorinated benzene complexes of simple cationic [Rh(Cy_2_P(CH_2_)_x_PCy_2_)]^+^ fragments rests on the concentrated reaction conditions—to overcome trace impurities found in commercially available arenes—and the use of the [Al{OC(CF_3_)_3_}_4_]^−^ anion—to avoid competition for metal binding by the anion. Of course, any weak arene binding observed is levelled by the bulk solvent used for further reactivity of these species. In instances where very weakly binding pro‐ligands or substrates are used, deploying a solvent such as F_5_C_6_H in conjunction with the appropriately modified operationally unsaturated precursor (e.g., **5 a**) could well prove to be a useful route for synthesis and catalysis.

## Experimental Section

All manipulations, unless otherwise stated, were performed under an argon atmosphere using standard Schlenk line and glovebox techniques. Glassware was oven dried at 130 °C overnight and flame dried under vacuum prior to use. Pentane and CH_2_Cl_2_ were dried using a Grubbs type solvent purification system (MBraun SPS‐800) and degassed by three successive freeze‐pump‐thaw cycles. Fluorinated benzenes (purchased from Fluorochem, pretreated with alumina) and CD_2_Cl_2_ were dried over CaH_2_, vacuum distilled, degassed by three successive freeze‐pump‐thaw cycles and stored over 3 Å molecular sieves. Norbornadiene (NBD) was dried over sodium, vacuum distilled, degassed by three successive freeze‐pump‐thaw cycles and stored over 3 Å molecular sieves. The synthesis of **1 a**, **1 b** and **2 b** are reported in the supporting information. Complexes **2 a** and **3 a** were prepared by the literature procedures.^13^ Li[Al{OC(CF_3_)_3_}_4_]14a, 28 and [Rh(NBD)Cl]_2_ 29 were prepared by modifications of the literature procedures. All other chemicals were obtained from commercial sources and used as received.

Solution NMR data were collected on either a Bruker Avance III HD nanobay NMR spectrometer equipped with a 9.4 T magnet or a Bruker Avance III NMR spectrometer equipped with a 11.75 T magnet at room temperature, unless otherwise stated. For samples collected in neat PFB the spectrometer was prelocked and shimmed to a mixture of C_6_D_6_ (25 %) and 1,2‐F_2_C_6_H_4_ (75 %) in a thick‐walled NMR tube. ^1^H NMR spectra collected in deuterated solvents were referenced to residual protio solvent. ^1^H NMR spectra collected in neat PFB were referenced to literature values^13^ or the center of the solvent resonance (1,2,3,4‐F_4_C_6_H_2_, *δ*=6.93; F_5_C_6_H, *δ*=6.92). ^31^P and ^19^F NMR spectra were referenced against 85 % H_3_PO_4_ (external) and CCl_3_F (external), respectively. Chemical shifts (δ) are quoted in ppm and coupling constants (*J*) in Hz. ESI‐MS were recorded on a Bruker MicrOTOF instrument interfaced with a glovebox.^30^ Microanalyses were performed by Stephen Boyer at London Metropolitan University.

**Synthesis of [Rh(NBD)_2_][Al{OC(CF_3_)_3_}_4_]**: An orange solution of [Rh(NBD)Cl]_2_ (458 mg, 0.993 mmol) in CH_2_Cl_2_ (10 mL) was added dropwise to a colorless slurry of Li[Al{OC(CF_3_)_3_}_4_] (1.95 g, 2.00 mmol) in CH_2_Cl_2_ (70 mL) and NBD (0.4 mL) with vigorous stirring at ambient temperature. The color of the slurry immediately changed to dark red. The reaction mixture was stirred at ambient temperature for a further 16 h and then filtered. The supernant was then concentrated under vacuum (ca. 50 mL). Cooling to −20 °C overnight afforded a red crystalline solid which was isolated by decanting, washed with pentane (2×2 mL) and dried under vacuum. Additional crops were obtained by further concentrating and cooling the supernant. Yield: 1.98 g (79 %). ^1^H NMR (CD_2_Cl_2_, 500 MHz): 5.64 (m, 8 H, alkene), 4.32 (s, 4 H, NBD bridgehead CH), 1.72 ppm (s, 4 H, NBD bridge CH_2_). ^19^F{^1^H} NMR (CD_2_Cl_2_, 470 MHz): *δ* −75.74 ppm (s, [Al{OC(CF_3_)_3_}_4_]). ESI‐MS found (calculated) *m*/*z=*287.03 (287.03). Elemental analysis found (calculated): C 28.51 (28.72), H 1.32 (1.29).

**Synthesis of [Rh(Cy_2_PCH_2_PCy_2_)(NBD)][Al{OC(CF_3_)_3_}_4_] (6 a)**: A solution of [Rh(NBD)_2_][Al{OC(CF_3_)_3_}_4_] (307 mg, 0.245 mmol) in CH_2_Cl_2_ (50 mL) was treated dropwise with a solution of Cy_2_PCH_2_PCy_2_ (100 mg, 0.245 mmol) in CH_2_Cl_2_ (20 mL) at ambient temperature with vigorous stirring. Upon complete addition the color of the reaction mixture changed from burgundy to orange. After 4 h, the solution was filtered and the solvent was then removed under vacuum. The resultant orange solid was washed with pentane and dried under vacuum. Yield: 305 mg (78 %). Crystals suitable for a single crystal X‐ray diffraction study were grown by layering a saturated CH_2_Cl_2_ solution with pentane. ^1^H NMR (CD_2_Cl_2_, 400 MHz): *δ* 5.70 (br s, 4 H, alkene), 4.22 (s, 2 H, NBD bridgehead CH), 3.04 (t, *J*_PH_=9.4 Hz, 2 H, PCH_2_P), 2.02–1.78 (m, 24 H, Cy), 1.72 (s, 2 H, NBD bridge CH_2_), 1.46–1.15 ppm (m, 20 H, Cy). ^31^P{^1^H} NMR (CD_2_Cl_2_, 162 MHz): δ −22.6 ppm (d, *J*_RhP_=133 Hz). ^19^F{^1^H} NMR (CD_2_Cl_2_, 376 MHz): δ−75.69 ppm (s, [Al{OC(CF_3_)_3_}_4_]). ESI‐MS found (calculated) *m*/*z=*603.25 (603.28). Elemental analysis found (calculated): C 36.81 (36.70), H 3.30 (3.47).

**Synthesis of [Rh(Cy_2_PCH_2_CH_2_PCy_2_)(NBD)][Al{OC(CF_3_)_3_}_4_] (6 b)**: A solution of [Rh(NBD)_2_][Al{OC(CF_3_)_3_}_4_] (465 mg, 0.37 mmol) in CH_2_Cl_2_ (60 mL) was treated dropwise with a solution of Cy_2_PCH_2_CH_2_PCy_2_ (156 mg, 0.37 mmol) in CH_2_Cl_2_ (30 mL) at ambient temperature with vigorous stirring. Upon complete addition the color of the reaction mixture changed from burgundy to orange. After 4 h, the solution was filtered and the solvent was then removed under vacuum. The resultant orange solid was washed with pentane and dried under vacuum. Yield: 540 mg (92 %). Crystals suitable for a single crystal X‐ray diffraction study were grown by layering a saturated CH_2_Cl_2_ solution with pentane. ^1^H NMR (CD_2_Cl_2_, 400 MHz): *δ* 5.54 (br s, 4 H, alkene), 4.20 (s, 2 H, NBD bridgehead CH), 2.06–1.70 (multiple aliphatic resonances, 30 H), 1.95–1.65 (multiple overlapping aliphatic resonances, 27 H), 1.39–1.19 (multiple overlapping aliphatic resonances, 16 H), 1.16–1.03 ppm (multiple aliphatic resonances, 4 H). ^31^P{^1^H} NMR (CD_2_Cl_2_, 162 MHz): *δ* 69.8 ppm (d, *J*_RhP_=154 Hz). ^19^F{^1^H} NMR (CD_2_Cl_2_, 376 MHz): δ−75.70 ppm (s, [Al{OC(CF_3_)_3_}_4_]). ESI‐MS found (calculated) *m*/*z=*617.29 (617.29). Elemental analysis found (calculated): C 37.08 (37.14), H 3.47 (3.56).

**General procedure for the preparation of η‐PFB complexes**: Complex **6 a** or **6 b** (50 mg, ≈31 μmol) was dissolved in PFB (0.15 mL) in a thick‐walled NMR tube fitted with a quick pressure valve. The solution was freeze‐pump‐thaw degassed thrice and refilled with H_2_ (4 atm, 298 K). The NMR tube was sealed and thoroughly mixed for ca. 2 minutes during which time the solution changed color from orange to golden yellow. The excess H_2_ was removed by freeze‐pump‐thaw degassing and the mixture was then characterized in situ by multinuclear NMR spectroscopy. The solution was filtered and layered with pentane to afford yellow crystals suitable for X‐ray diffraction which were isolated by filtration and dried under vacuum.

**5 a [Rh(Cy_2_PCH_2_PCy_2_)(η^6^‐F_5_C_6_H)][Al{OC(CF_3_)_3_}_4_]**: Yield: 32 mg (61 %). ^1^H NMR (F_5_C_6_H, 500 MHz): *δ* 7.29 (br s, 1 H, η‐F_5_C_6_H), 3.10 (t, *J*_PH_=9.8 Hz, 2 H, PCH_2_P), 2.26–2.02 (multiple overlapping aliphatic resonances, 20 H), 1.66–1.32 ppm (multiple overlapping aliphatic resonances, 24 H). ^31^P{^1^H} NMR (F_5_C_6_H, 202 MHz): *δ* −12.4 ppm (d, *J*_RhP_=164 Hz). ^19^F{^1^H} NMR (F_5_C_6_H, 470 MHz): *δ* −76.78 (s, 36 F, [Al{OC(CF_3_)_3_}_4_]), −149.77 (br apparent d, *J*_FF_=29 Hz, 2 F, η‐1,5‐F_5_C_6_H), −165.00 (br apparent t, *J*_FF_=31 Hz, 1 F, η‐3‐F_5_C_6_H), −167.76 ppm (apparent br t, *J*_FF_=30 Hz, 2 F, η‐2,4‐F_5_C_6_H). Elemental analysis found (calculated): C 34.15 (34.28), H 2.93 (2.88).

**5 b [Rh(Cy_2_PCH_2_CH_2_PCy_2_)(η^6^‐F_5_C_6_H)][Al{OC(CF_3_)_3_}_4_]**: Yield: 26 mg (49 %). ^1^H NMR (F_5_C_6_H, 500 MHz): *δ* 7.19 (br s, 1 H, η‐F_5_C_6_H), 2.28–1.92 (multiple overlapping aliphatic resonances, 24 H), 1.60–1.33 ppm (multiple overlapping aliphatic resonances, 24 H). ^31^P{^1^H} NMR (F_5_C_6_H, 202 MHz): *δ* 97.8 ppm (d, *J*_RhP_=193 Hz). ^19^F{^1^H} NMR (F_5_C_6_H, 470 MHz): *δ* −76.81 (s, 36 F, [Al{OC(CF_3_)_3_}_4_]), −148.68 (br d, *J*_FF_=29 Hz, 2 F, η‐1,5‐F_5_C_6_H), −163.87 (br t, *J*_FF_=31 Hz, 1 F, η‐3‐F_5_C_6_H), −166.64 ppm (apparent br t, *J*_FF_=30 Hz, 2 F, η‐2,4‐F_5_C_6_H). Elemental analysis found (calculated): C 34.57 (34.72), H 2.69 (2.97).

**4 a [Rh(Cy_2_PCH_2_PCy_2_)(η^6^‐F_4_C_6_H_2_)][Al{OC(CF_3_)_3_}_4_]**: Yield: 29 mg (56 %). ^1^H NMR (1,2,3,4‐F_4_C_6_H_2_, 500 MHz): *δ* 3.07 (t, J_PH_=10 Hz, 2 H, PCH_2_P), 2.20–1.99 (multiple overlapping aliphatic resonances, 20 H), 1.62–1.31 (multiple overlapping aliphatic resonances, 24 H). The ^1^H resonance of η‐F_4_C_6_H_2_ is presumably obscured by the solvent. ^31^P{^1^H} NMR (1,2,3,4‐F_4_C_6_H_2_, 202 MHz): *δ* −11.9 ppm (d, *J*_RhP_=166 Hz). ^19^F{^1^H} NMR (1,2,3,4‐F_4_C_6_H_2_, 470 MHz): *δ* −76.33 (s, 36 F, [Al{OC(CF_3_)_3_}_4_]), −149.48 (br apparent d, *J*_FF_=26 Hz, 2 F, η‐1,4‐F_4_C_6_H_2_), −164.98 ppm (m, 2 F, η‐2,3‐F_4_C_6_H_2_). Elemental analysis found (calculated): C 34.72 (34.66), H 3.08 (2.97).

**4 b [Rh(Cy_2_PCH_2_CH_2_PCy_2_)(η^6^‐F_4_C_6_H_2_)][Al{OC(CF_3_)_3_}_4_]**: Yield: 29 mg (55 %). ^1^H NMR (1,2,3,4‐F_4_C_6_H_2_, 500 MHz): *δ* 2.20–1.80 (multiple overlapping aliphatic resonances, 24 H), 1.58–1.31 (multiple overlapping aliphatic resonances, 24 H). The ^1^H resonance of η‐F_4_C_6_H_2_ is presumably obscured by the solvent. ^31^P{^1^H} NMR (1,2,3,4‐F_4_C_6_H_2_, 202 MHz): *δ* 97.9 ppm (d, *J*_RhP_=195 Hz). ^19^F{^1^H} NMR (1,2,3,4‐F_4_C_6_H_2_, 470 MHz): *δ* −76.36 (s, 36 F, [Al{OC(CF_3_)_3_}_4_]), −148.98 (apparent br d, *J*_FF_=25 Hz, 2 F, 1,4‐F_4_C_6_H_2_), −163.91 ppm (m, 2 F, 2,3‐F_4_C_6_H_2_). Elemental analysis found (calculated): C 34.86 (35.10), H 2.94 (3.07).

**3 b [Rh(Cy_2_PCH_2_CH_2_PCy_2_)(η^6^‐F_3_C_6_H_3_)][Al{OC(CF_3_)_3_}_4_]**: Yield: 38 mg (73 %). ^1^H NMR (1,2,3‐F_3_C_6_H_3_, 500 MHz): *δ* 6.63 (br s, 3 H, η‐F_3_C_6_H_3_), 2.12–1.70 (multiple overlapping aliphatic resonances, 24 H), 1.47–1.17 (multiple overlapping aliphatic resonances, 24 H). ^31^P{^1^H} NMR (1,2,3‐F_3_C_6_H_3_, 202 MHz): *δ* 98.3 ppm (d, J_RhP_=198 Hz). ^19^F{^1^H} NMR (1,2,3‐F_3_C_6_H_3_, 470 MHz): δ−75.96 (s, 36 F, [Al{OC(CF_3_)_3_}_4_]), −146.27 (dd, *J*_FF_=30 Hz, *J*_RhF_=4.6 Hz, 2 F, 1,3‐F_3_C_6_H_3_,), −166.27 ppm (td, J_FF_=30 Hz, *J*_RhF_=5 Hz, 1 F, 2‐F_3_C_6_H_3_). Elemental analysis found (calculated): C 35.60 (35.48), H 3.15 (3.16).

**7 [Rh(Cy_2_PCH_2_CH_2_PCy_2_)(C_4_H_8_)][Al{OC(CF_3_)_3_}_4_]**: An in situ prepared solution of **5 b** (≈31.6 μmol) in F_5_C_6_H (0.15 mL) in a thick‐walled NMR tube fitted with a quick pressure valve, was freeze‐pump‐thawed degassed thrice and refilled with isobutene (1 atm, 298 K). The vessel was sealed and thoroughly mixed. The resultant mixture was filtered then layered with pentane at room temperature. This afforded light orange crystals suitable for X‐ray diffraction which were isolated by filtration and dried under vacuum. Yield: 25 mg (51 %). ^1^H NMR (CD_2_Cl_2_, 500 MHz, 193 K): *δ* 4.25 (br s, 1 H, alkene), 3.32 (br s, 1 H, alkene), 2.08–1.45 (multiple overlapping aliphatic resonances, 31 H), 1.31–0.92 (multiple overlapping aliphatic resonances, 20 H), −0.29 ppm (br s, 3 H, agostic CH_3_). ^31^P{^1^H} NMR (CD_2_Cl_2_, 202 MHz, 193 K): *δ* 97.5 ppm (dd, *J*_RhP_ 202 Hz, *J*_PP_ 25 Hz), 93.6 ppm (dd, *J*_RhP_ 158 Hz, *J*_PP_ 25 Hz). ^19^F{^1^H} NMR (CD_2_Cl_2_, 470 MHz, 193 K): *δ* −75.91 ppm (s, [Al{OC(CF_3_)_3_}_4_]). Elemental analysis found (calculated): C 34.56 (35.67), H 2.95 (3.65).

**Tishchenko reactivity of cyclohexanecarboxaldehyde**: To an in situ prepared solution of [Rh(Cy_2_PCH_2_PCy_2_)(F_n_C_6_H_6−*n*_)][Al{OC(CF_3_)_3_}_4_] (≈35 μmol) in PFB (0.35 mL) was added a stock solution of cyclohexanecarboxaldehyde (85 μL, 0.70 mmol) and the reaction mixture shaken vigorously before being monitored by ^1^H NMR spectroscopy. The reaction crude was purified by silica gel chromatography (Petrol/Et_2_O, 8:2) affording cyclohexylmethyl cyclohexanecarboxylate as a colorless oil. ^1^H NMR (CDCl_3_, 400 MHz): *δ* 3.86 (d, *J*_HH_=6.5 Hz, 2 H), 2.31 (tt, *J*_HH_=11.0 Hz, 3.5 Hz, 1 H), 1.92–1.86 (m, 2 H), 1.74–1.64 (m, 9 H), 1.46–1.35 (m, 2 H), 1.34–1.15 (m, 6 H), 0.98–0.91 ppm (m, 2 H). ^13^C{^1^H} NMR (CDCl_3_, 101 MHz): *δ* 176.3, 69.4, 43.4, 37.3, 29.8, 29.2, 25.9, 25.8, 25.6 ppm. LRMS (ESI^+^) *m*/*z* 225.1 [*M*+H]^+^. Data are consistent with that reported in the literature.^27^

## Conflict of interest

The authors declare no conflict of interest.

## Supporting information

As a service to our authors and readers, this journal provides supporting information supplied by the authors. Such materials are peer reviewed and may be re‐organized for online delivery, but are not copy‐edited or typeset. Technical support issues arising from supporting information (other than missing files) should be addressed to the authors.

Supplementary
