# Supplementary material for: Synthesis of Highly Fluorinated Arene Complexes of [Rh(Chelating Phosphine)]+ Cations, and their use in Synthesis and Catalysis
Source: Chemistry. 2020 Feb 11;26(13):2883–9. doi: 10.1002/chem.201904668 (PMC7078928; doi:10.1002/chem.201904668)
Supplement: Supplementary file 1 — Supplementary [file CHEM-26-2883-s001.pdf]

CHEMISTRY
A European Journal

Supporting Information

Synthesis of Highly Fluorinated Arene Complexes of [Rh(Chelating Phosphine)]$^{+}$ Cations, and their use in Synthesis and Catalysis

Alasdair I. McKay,[a] James Barwick-Silk,[a] Max Savage,[a] Michael C. Willis,[a] and Andrew S. Weller*[a, b]

chem_201904668_sm_miscellaneous_information.pdf

TABLE OF CONTENTS

S.1. FURTHER SYNTHESIS 2
S.1.1. General procedure for the synthesis of fluorobenzene and 1,2-difluorobenzene complexes 2
S.1.1.1. Characterization data for 1a 2
S.1.1.2. Characterization data for 1b 2
S.1.1.3. Characterization data for 2b 3
S.2. NMR SPECTRA 4
S.3. CRYSTALLOGRAPHIC AND REFINEMENT DATA 30
S.3.1. Crystal structure determinations 30
S.3.2. Further comments on crystal structures 30
S.3.3. Further crystal structures 31
S.4. REFERENCES 36

S.1. Further Synthesis

S.1.1. General procedure for the synthesis of fluorobenzene and 1,2-difluorobenzene complexes

A J. Young's flask was charged with $[Rh(Cy_2P(CH_2)_xPCy_2)(COD)][Al\{OC(CF_3)_3\}_4]$ (x = 1 or 2).[S1] Fluoroarene (50 mL/mmol) was then added and the resultant orange solution was freeze-pump-thaw degassed three times and backfilled with H_2 (4 bar). The reaction mixture was stirred for a further 16 h at ambient temperature. During which time the color of solution changed to yellow. The solvent and excess H_2 were then removed in vacuo. The resultant solid was washed with pentane (2 × 5 mL) before being extracted into the minimum volume of CH_2Cl_2 and filtered. Layering with pentane afforded single crystals suitable for an X-ray diffraction study.

S.1.1.1. Characterization data for 1a

Yield: 33 mg (67%). ^{1}H NMR (CD_2Cl_2, 400 MHz): δ 6.74 (m, 2H, ortho-C_6H_5F), 6.64 (m, 2H, meta-C_6H_5F), 6.05 (m, 1H, para-C_6H_5F), 2.67 (td, J_{PH} = 10 Hz, J_{RhH} = 2 Hz, 2H, PCH_2P), 1.94-1.59 (br m, 24H, Cy), 1.39-1.02 (br m, 20H, Cy). ^{31}P{^{1}H} NMR (CD_2Cl_2, 162 MHz): δ -9.92(d, J_{RhP} = 170Hz). ^{19}F{^{1}H} NMR (CD_2Cl_2, 376 MHz): δ -75.76 (s, 36F, CF_3), -123.07 (d, J_{RhF} = 4 Hz, 1F, C_6H_5F). ESI-MS found (calculated) m/z = 607.26 (607.25). Elemental analysis found (calculated): C 36.01 (35.85), H 3.20 (3.26).

S.1.1.2. Characterization data for 1b

Yield: 55 mg (75%). ^{1}H NMR (CD_2Cl_2, 500 MHz): δ 6.72 (m, 2H, ortho-FC_6H_5), 6.63 (m, 2H, meta-FC_6H_5), 6.15 (m, 1H, para-C_6FH_5), 1.98-1.55 (multiple overlapping aliphatic resonances, 28H), 1.40-0.96 (multiple overlapping aliphatic resonances, 20H). ^{31}P{^{1}H} NMR (CD_2Cl_2, 202 MHz): δ 98.5 (d, J_{RhP} = 201 Hz). ^{19}F{^{1}H} NMR (CD_2Cl_2, 470 MHz): δ -75.73 (s, 36F, $[Al\{OC(CF_3)_3\}_4]$), -122.79 (s, 1F, FC_6H_5). ESI-MS found (calculated) m/z = 621.26 (621.27). Elemental analysis found (calculated): C 36.34 (36.29), H 3.30 (3.36).

S.1.1.3. Characterization data for 2b

Yield: 150 mg (72%). ^{1}H NMR (CD_2Cl_2, 400 MHz): δ 6.85 (m, 2H, $F_2C_6H_4$), 6.26 (m, 2H, $F_2C_6H_4$), 1.96-1.58 (multiple overlapping aliphatic resonances, 28H), 1.41-1.11 (multiple overlapping aliphatic resonances, 16H), 1.08-0.96 (multiple overlapping aliphatic resonances, 4H). ^{31}P{^{1}H} NMR (CD_2Cl_2, 162 MHz): δ 98.6 (d, J_{RhP} = 199 Hz). ^{19}F{^{1}H} NMR (CD_2Cl_2, 376 MHz): δ -75.73 (s, 36F, [Al{OC(CF_3)$_3$}$_4$]), -145.62 (d, J_{RhF} = 4 Hz, 2F, $F_2C_6H_4$). ESI-MS found (calculated) m/z = 639.26 (639.26). Elemental analysis found (calculated): C 35.97 (35.88), H 3.14 (3.26).

S.2. NMR Spectra

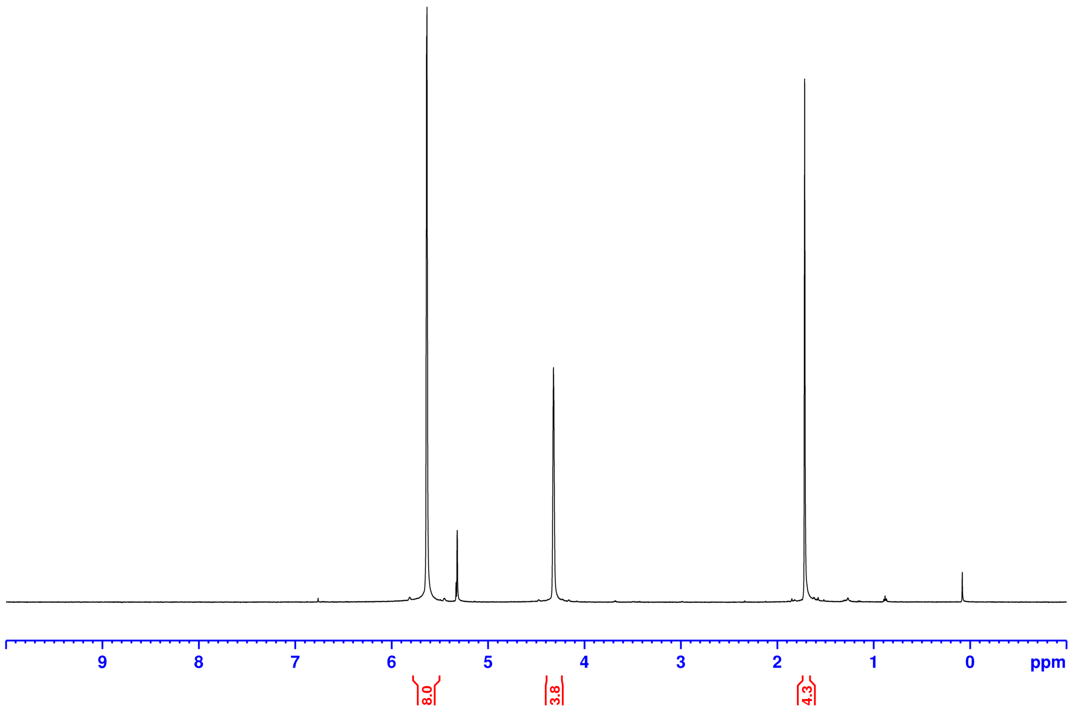

Figure S1: The ^{1}H NMR (CD_2Cl_2, 500 MHz) spectrum of **[Rh(NBD)$_2$][Al{OC(CF_3)$_3$}$_4$]**.

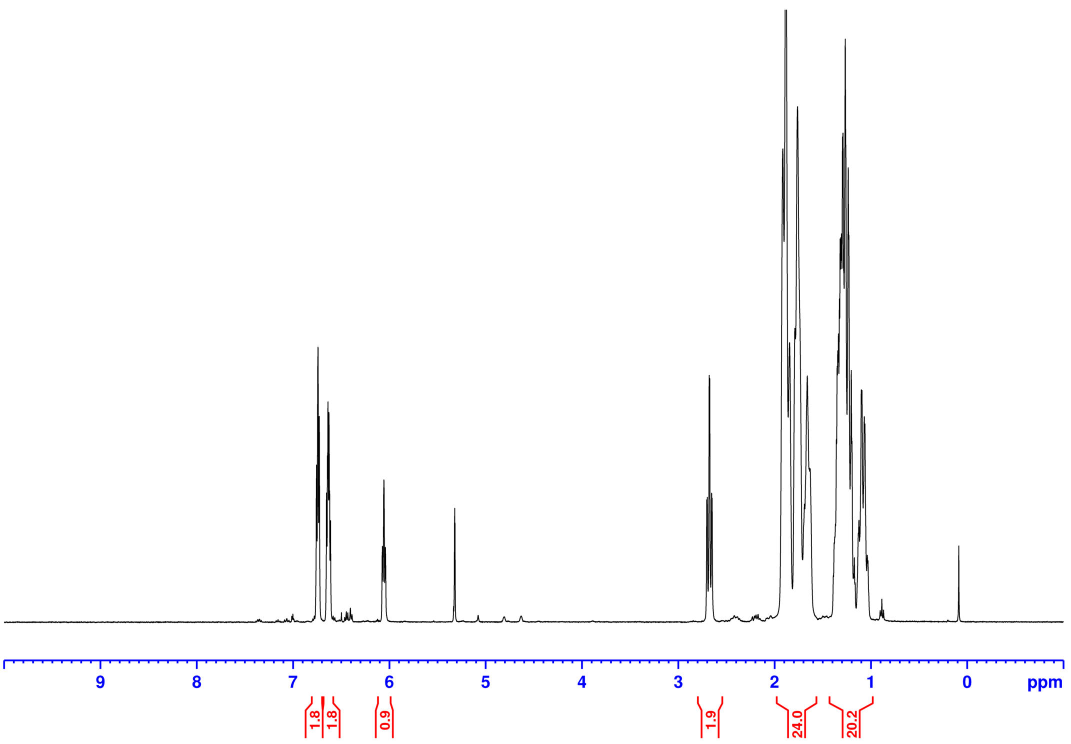

Figure S2: The ^{1}H NMR (CD_2Cl_2, 400 MHz) spectrum of **1a**.

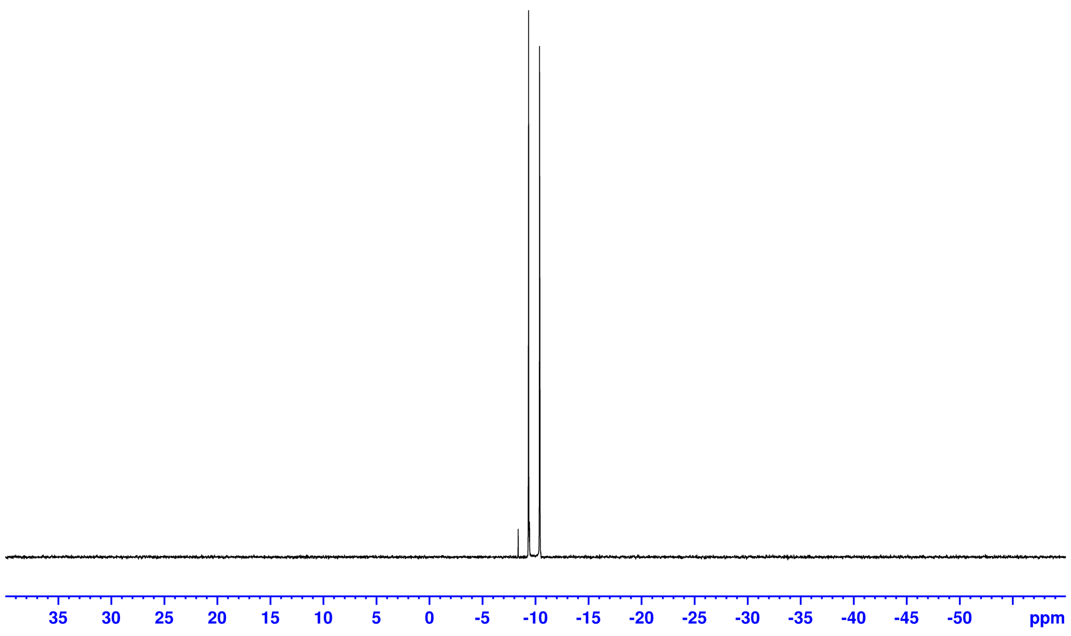

Figure S3: The $^{31}P\{^{1}H\}$ NMR (CD_2Cl_2, 162 MHz) spectrum of **1a**.

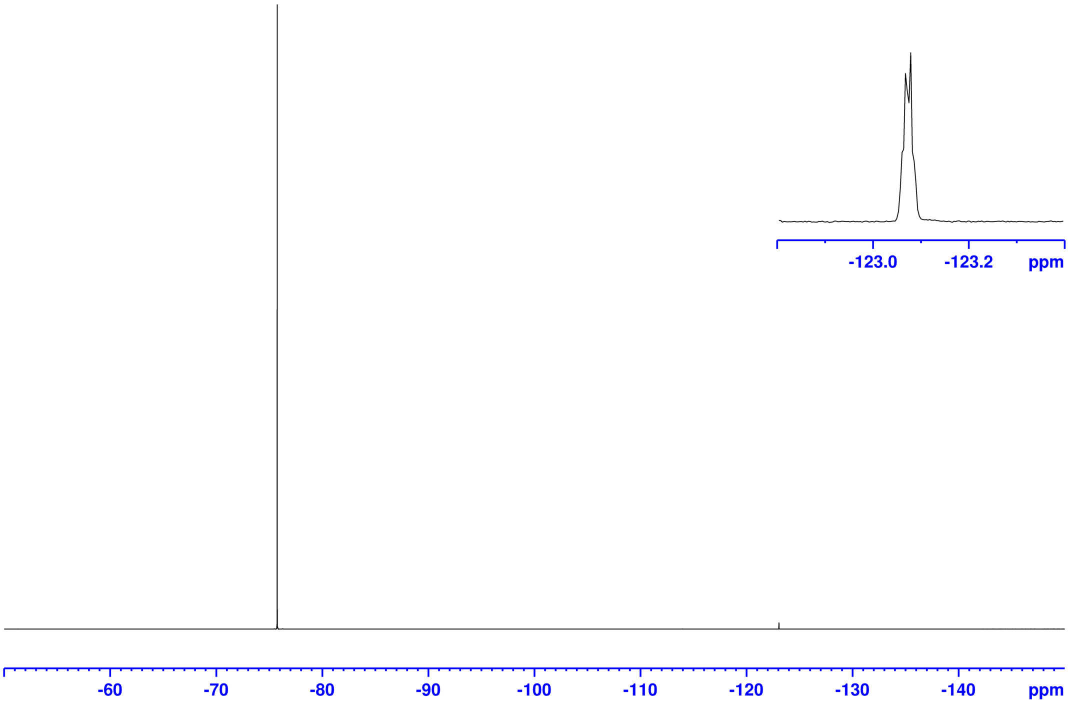

Figure S4: The $^{19}F\{^1H\}$ NMR (CD_2Cl_2, 376 MHz) spectrum of **1a**. The inset is an enlargement of the FC_6H_5 resonance.

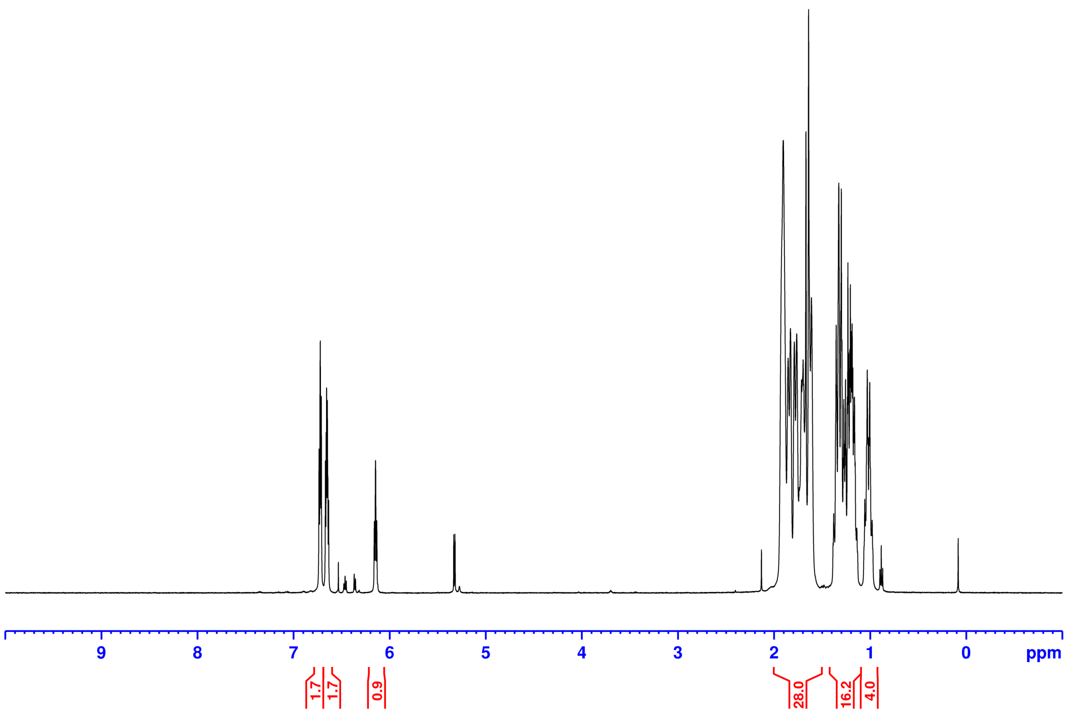

Figure S5: The ^{1}H NMR (CD_2Cl_2, 500 MHz) spectrum of **1b**.

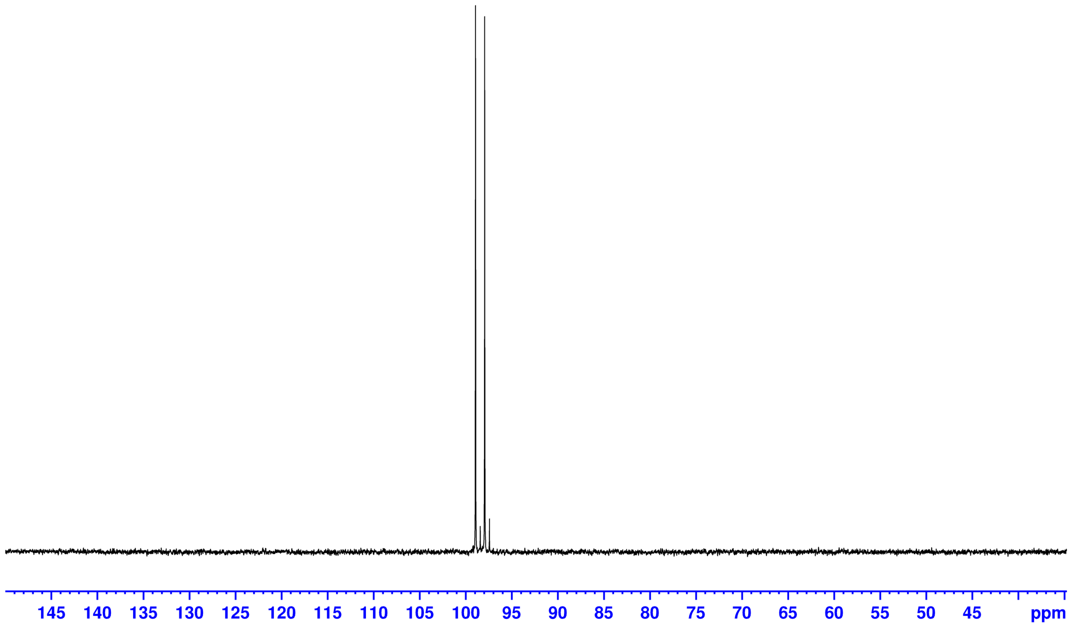

Figure S6: The $^{31}P\{^{1}H\}$ NMR (CD_2Cl_2, 202 MHz) spectrum of **1b**.

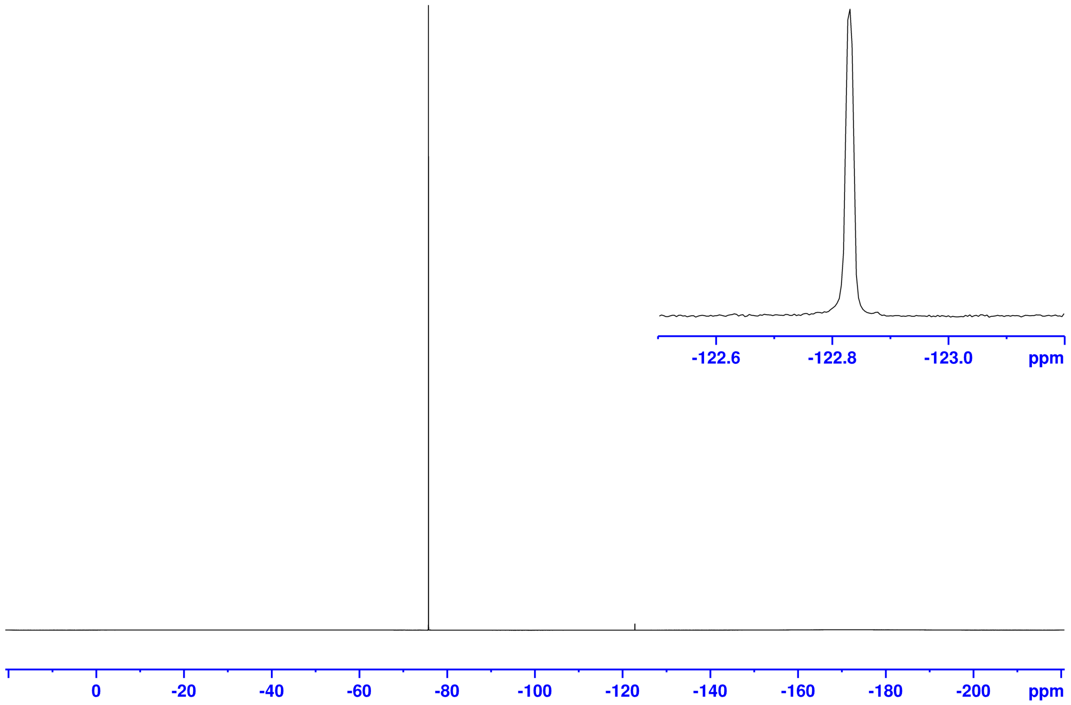

Figure S7: The $^{19}F\{^1H\}$ NMR (CD_2Cl_2, 470 MHz) spectrum of **1b**. The inset is an enlargement of the FC_6H_5 resonance.

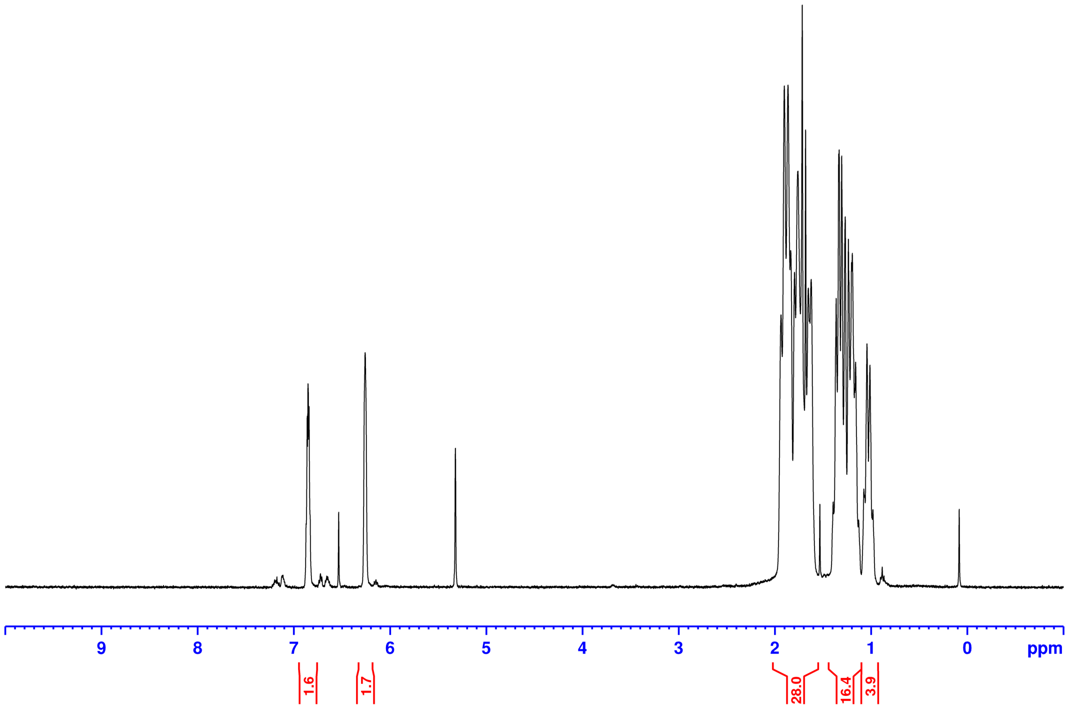

Figure S8: The ^{1}H NMR (CD_2Cl_2, 400 MHz) spectrum of **2b**.

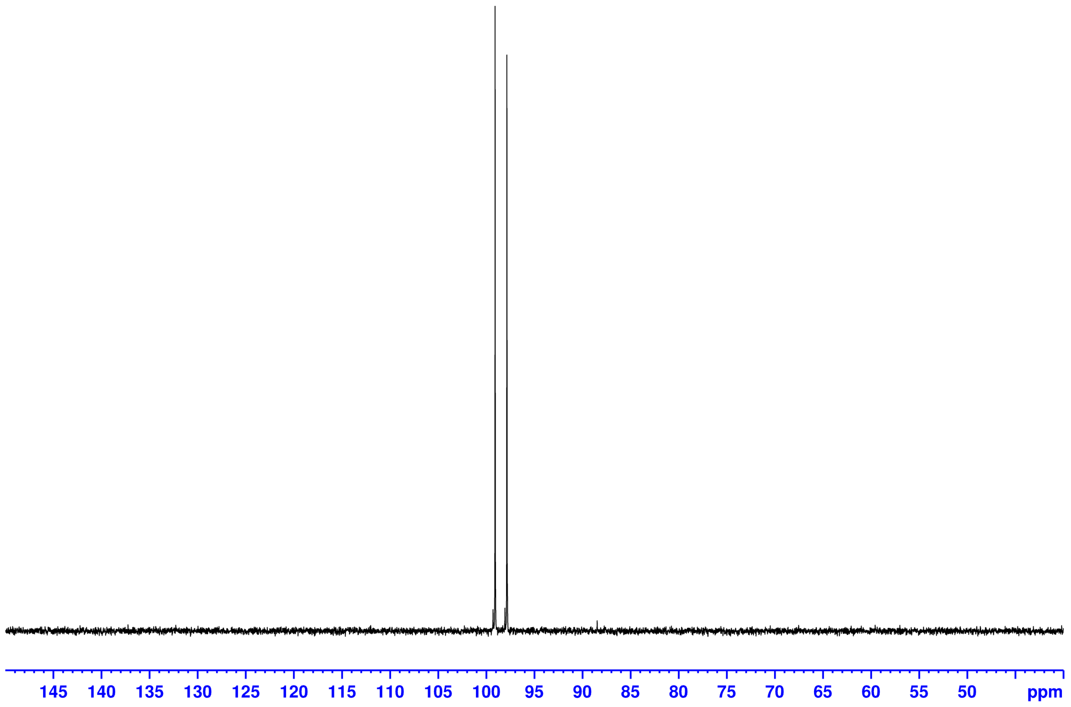

Figure S9: The ^{31}P{^{1}H} NMR (CD_2Cl_2, 162 MHz) spectrum of **2b**.

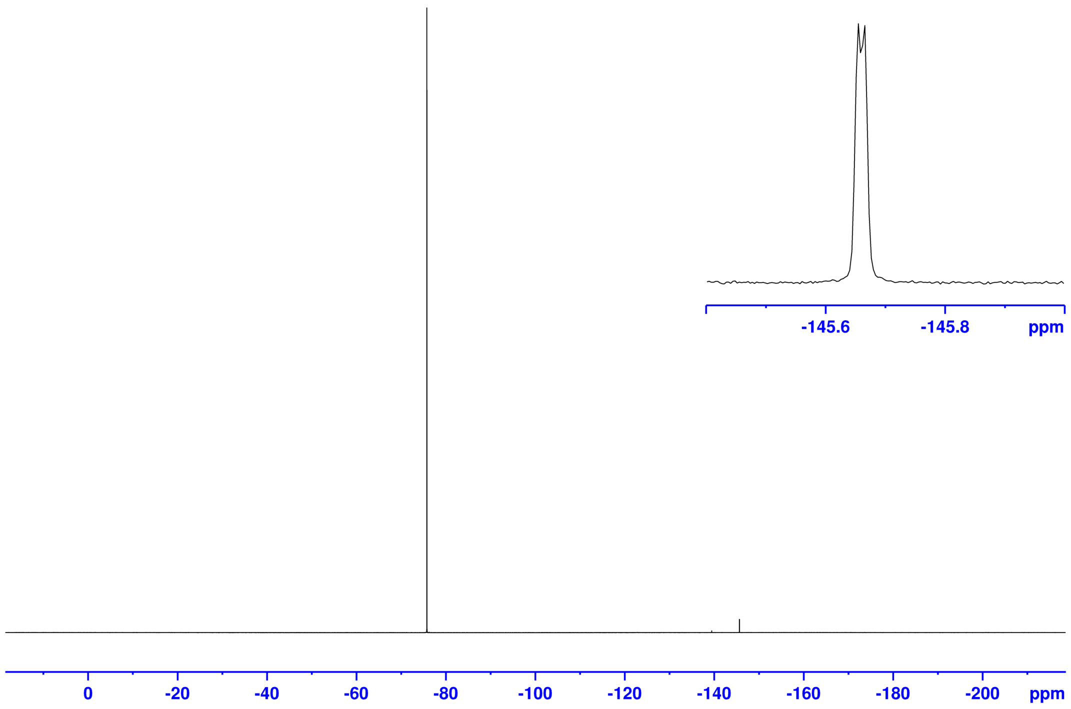

Figure S10: The $^{19}F\{^1H\}$ NMR (CD_2Cl_2, 376 MHz) spectrum of **2b**. The inset is an enlargement of the bound $F_2C_6H_4$ resonance.

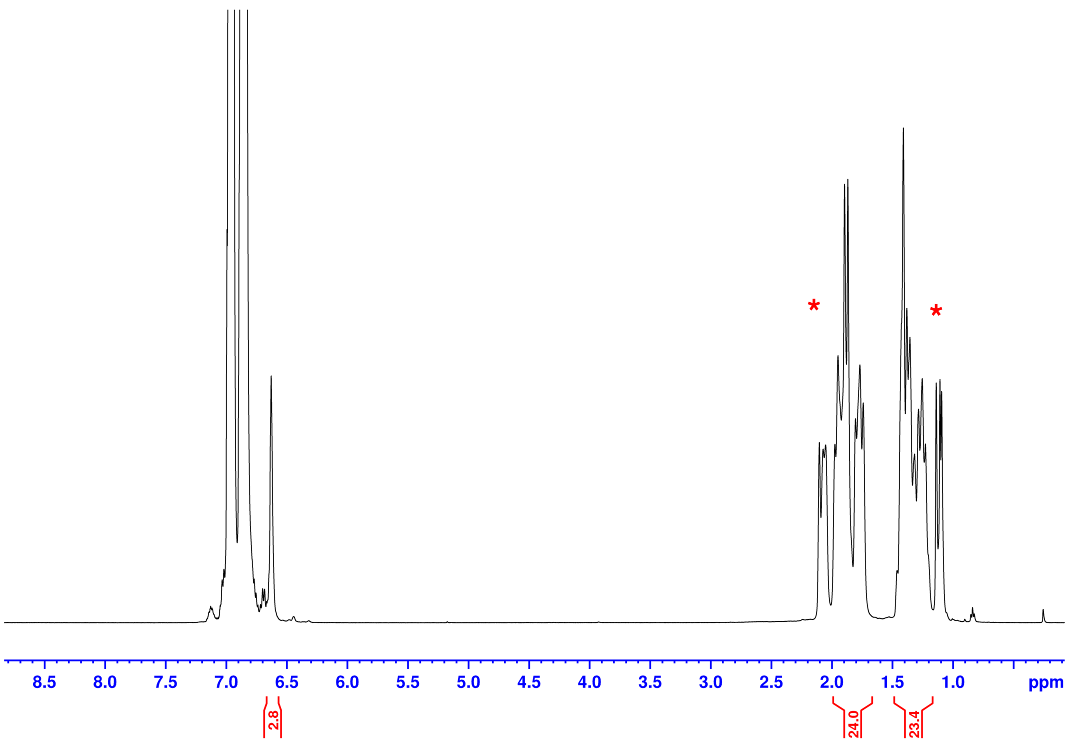

Figure S11: The ^{1}H NMR ($F_3C_6H_3$, 500 MHz) spectrum of **3b**. The red asterisks denote resonances for the NBA byproduct.

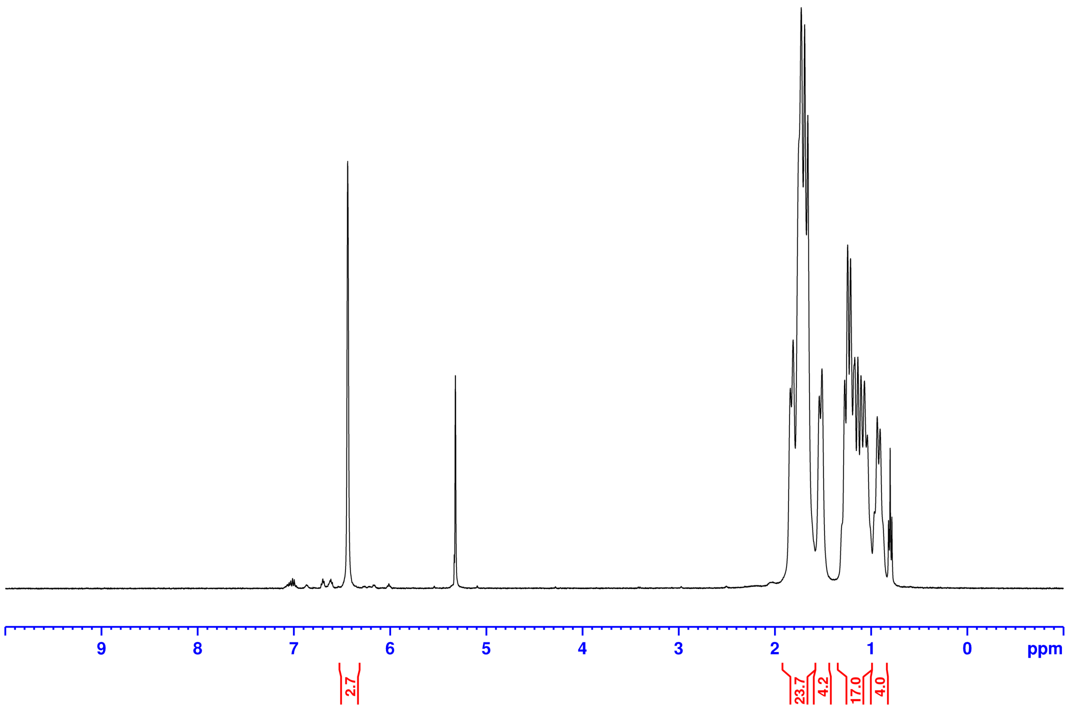

Figure S12: The ^{1}H NMR (CD_2Cl_2, 193 K, 400 MHz) spectrum of **3b**.

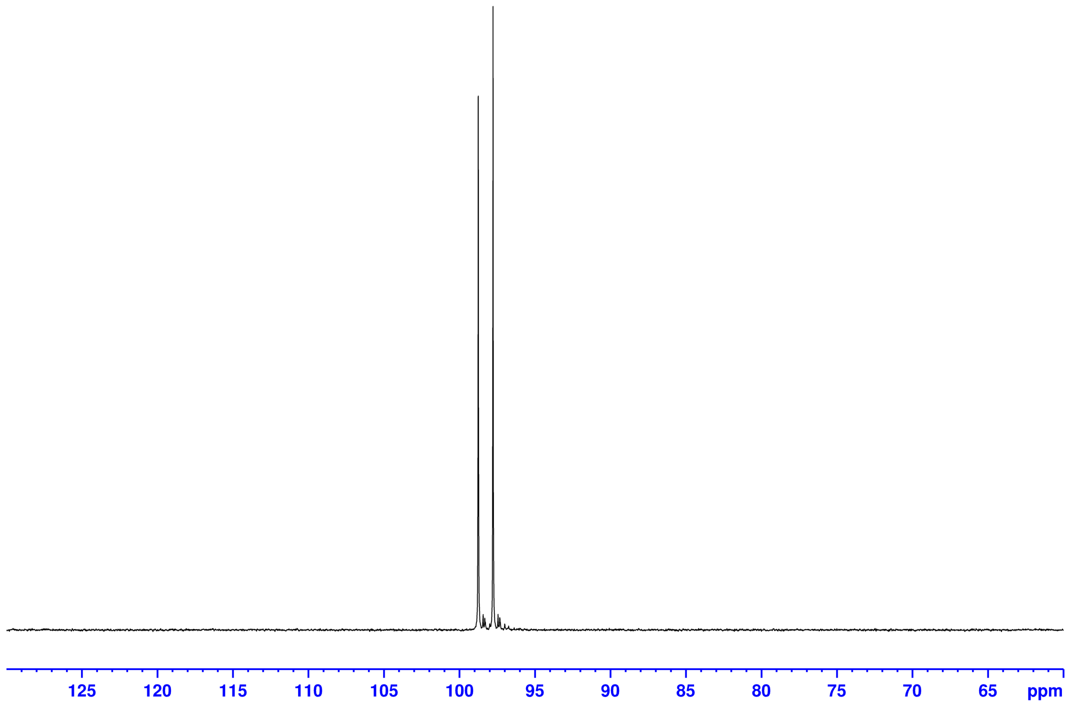

Figure S13: The $^{31}P\{^{1}H\}$ NMR ($F_3C_6H_3$, 202 MHz) spectrum of **3b**.

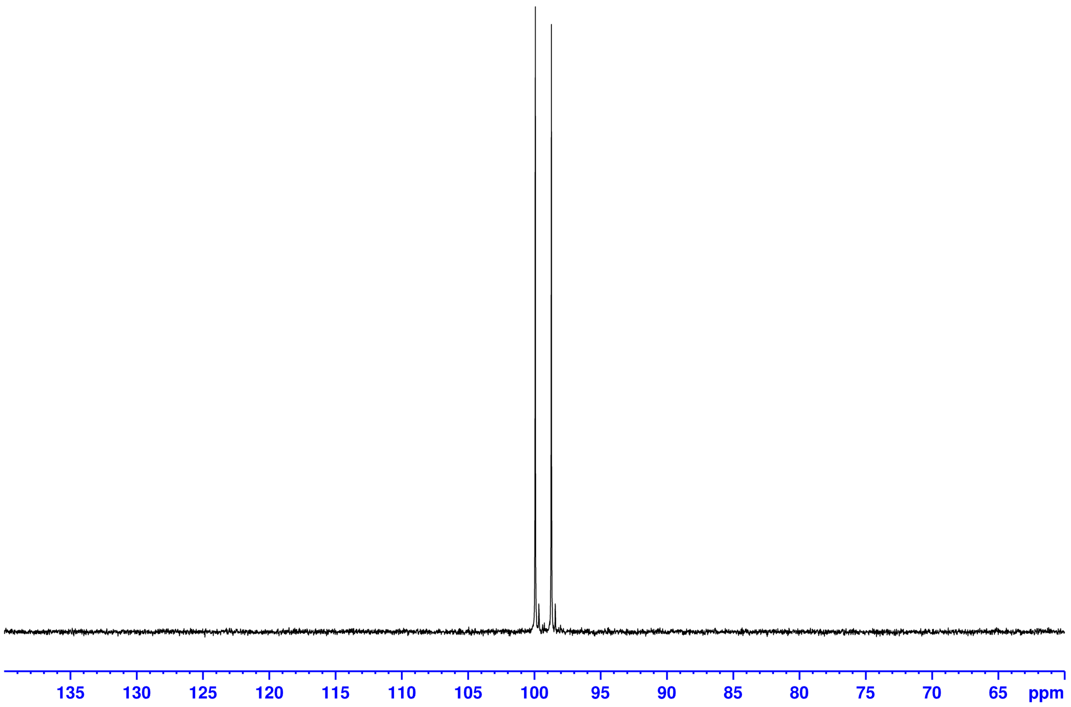

Figure S14: The $^{31}P\{^{1}H\}$ NMR (CD_2Cl_2, 193 K, 162 MHz) spectrum of **3b**.

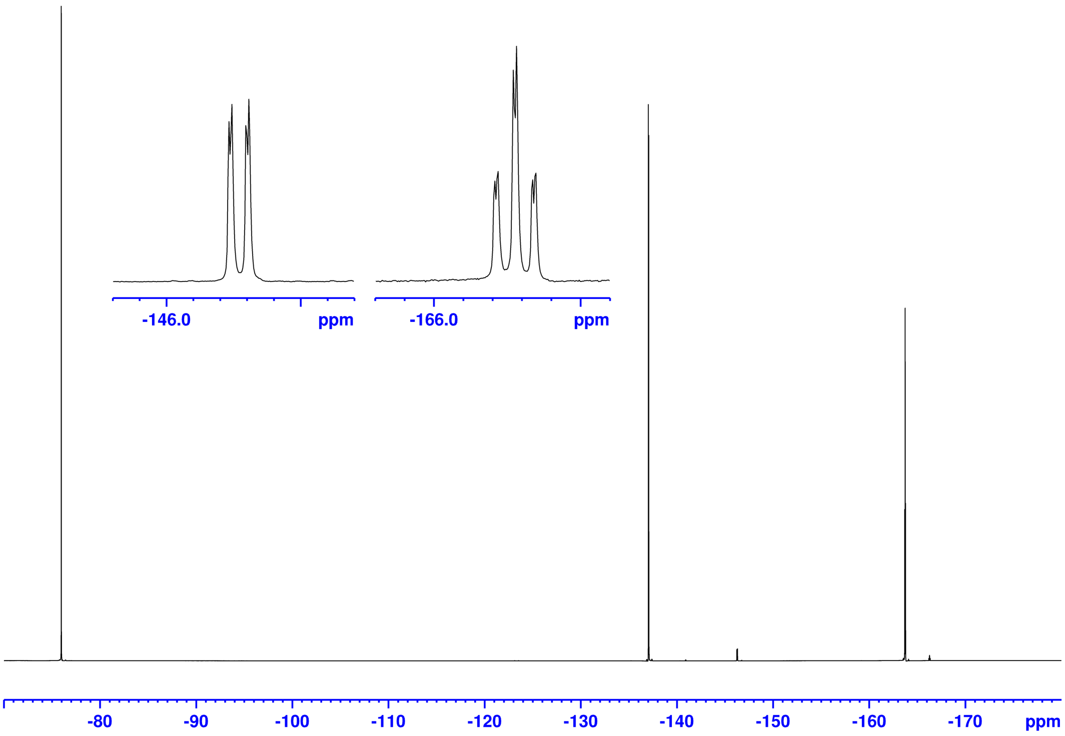

Figure S15: The ^{19}F{^{1}H} NMR ($F_3C_6H_3$, 470 MHz) spectrum of **3b**. The insets are enlargements of the bound $F_3C_6H_3$ resonances.

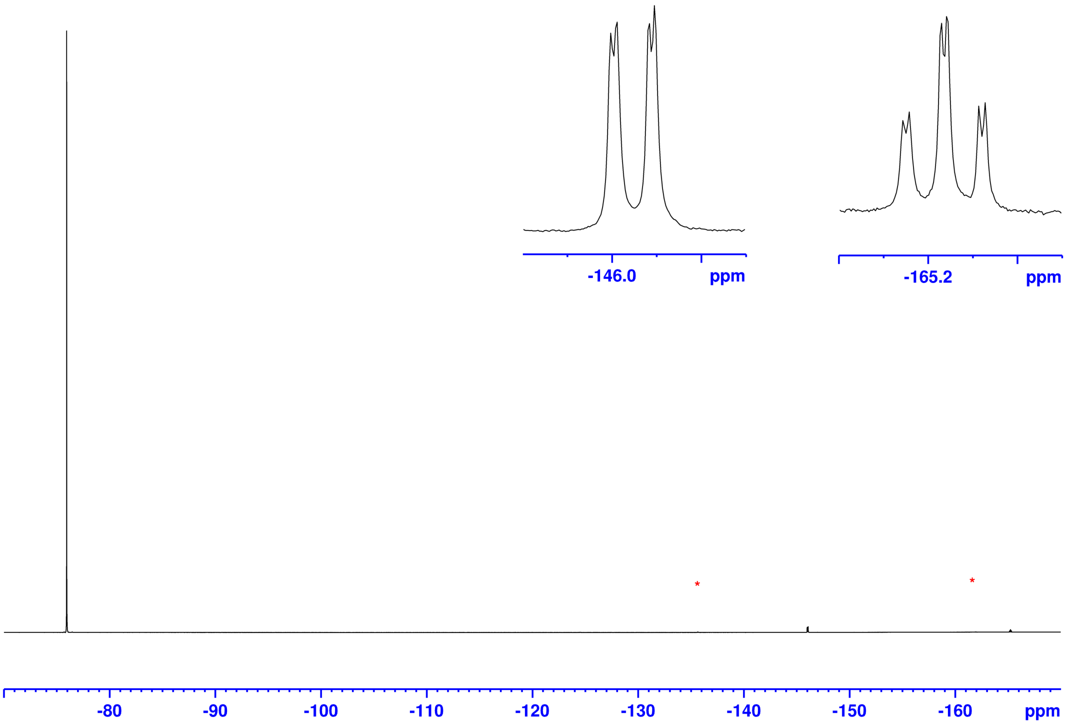

Figure S16: The ^{19}F{^{1}H} NMR (CD_2Cl_2, 193 K, 376 MHz) spectrum of **3b**. Red asterisks denote trace free $F_3C_6H_3$. The insets are enlargements of the bound $F_3C_6H_3$ resonances.

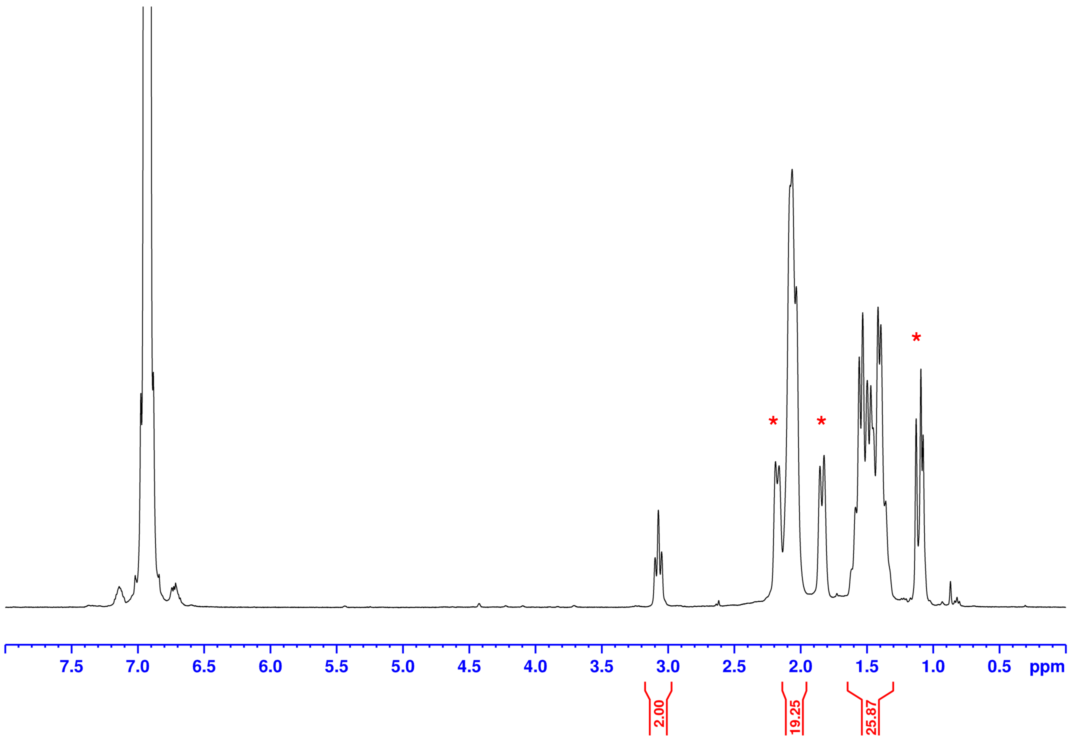

Figure S17: The ^{1}H NMR ($F_4C_6H_2$, 400 MHz) spectrum of **4a**. The red asterisks denote resonances for the NBA byproduct.

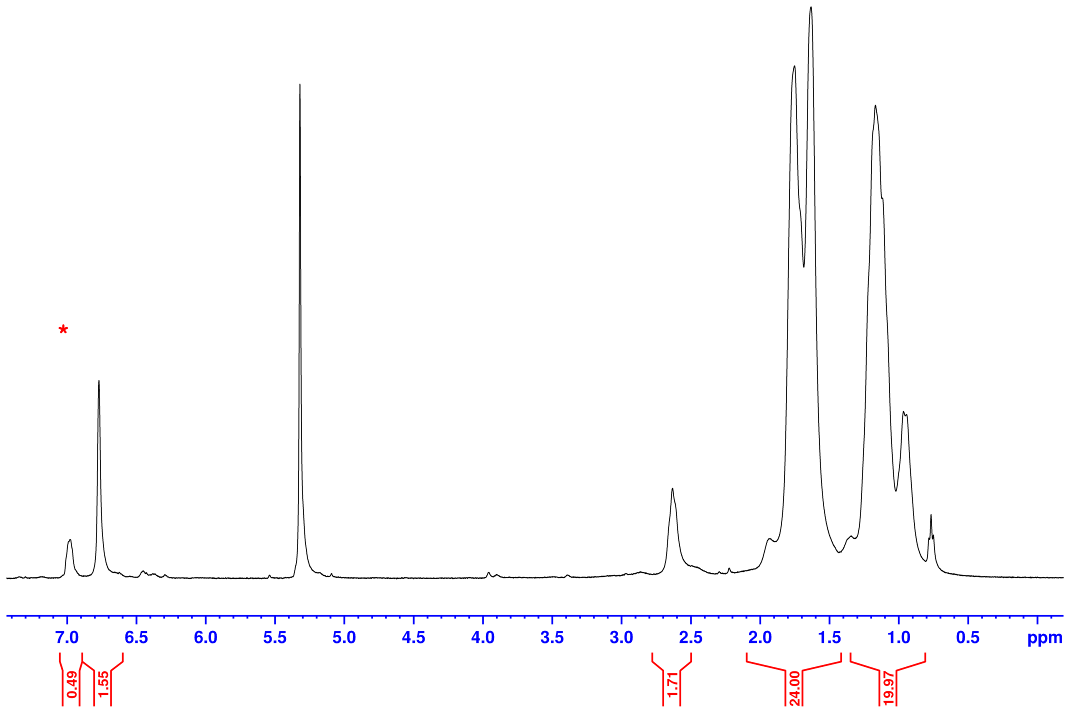

Figure S18: The ^{1}H NMR (CD_2Cl_2, 183 K, 400 MHz) spectrum of **4a**. The red asterisk denotes free $F_4C_6H_2$ potentially signaling partial decomposition (ca. 25%).

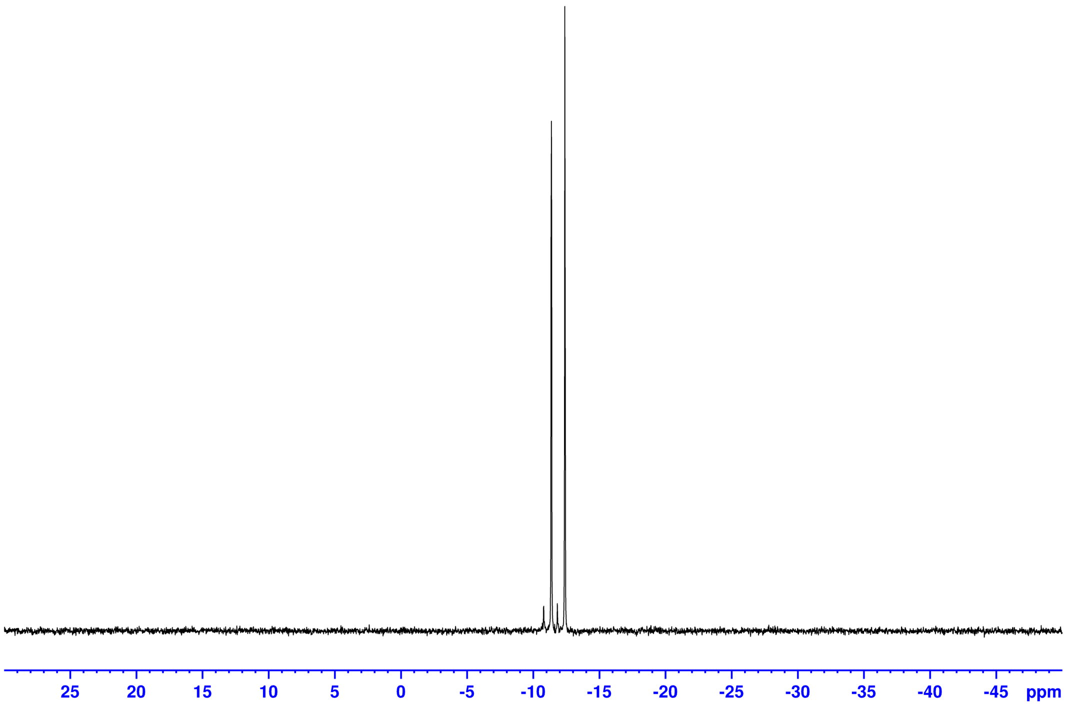

Figure S19: The $^{31}P\{^1H\}$ NMR ($F_4C_6H_2$, 162 MHz) spectrum of **4a**.

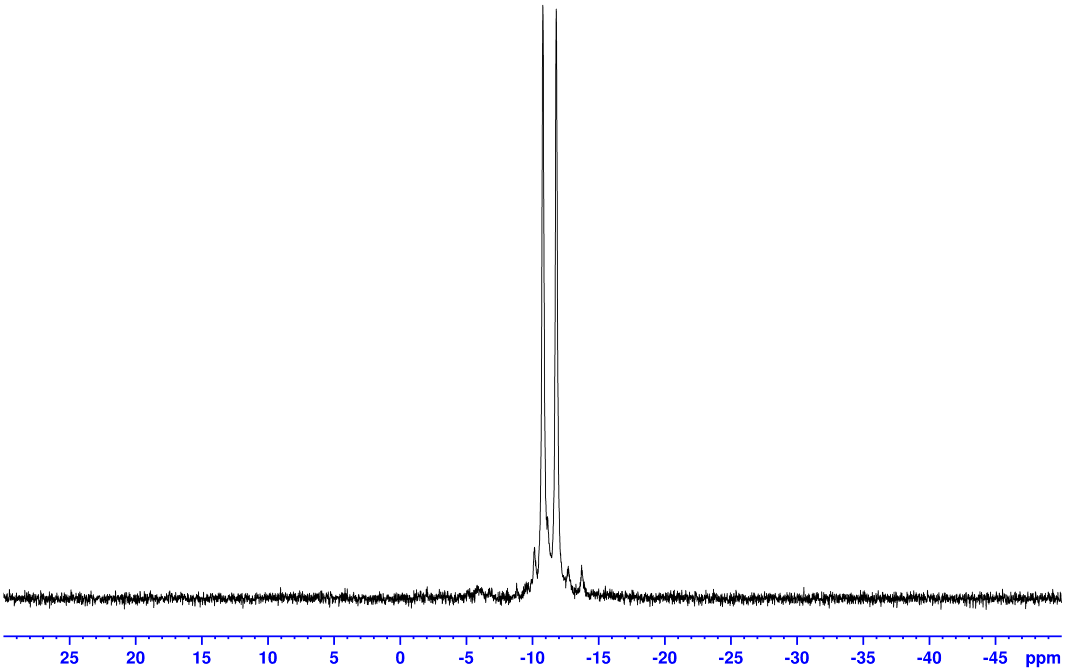

Figure S20: The $^{31}P\{^1H\}$ NMR (CD_2Cl_2, 183 K, 162 MHz) spectrum of **4a**.

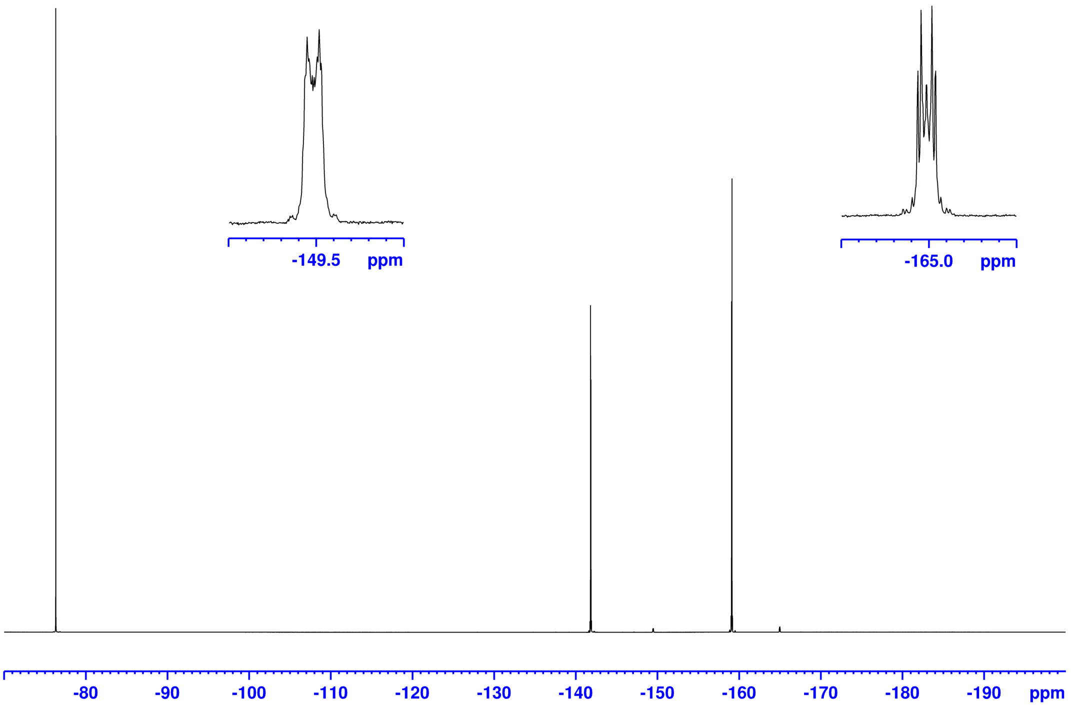

Figure S21: The $^{19}F\{^1H\}$ NMR ($F_4C_6H_2$, 376 MHz) spectrum of **4a**. The insets are enlargements of the bound $F_4C_6H_2$ resonances.

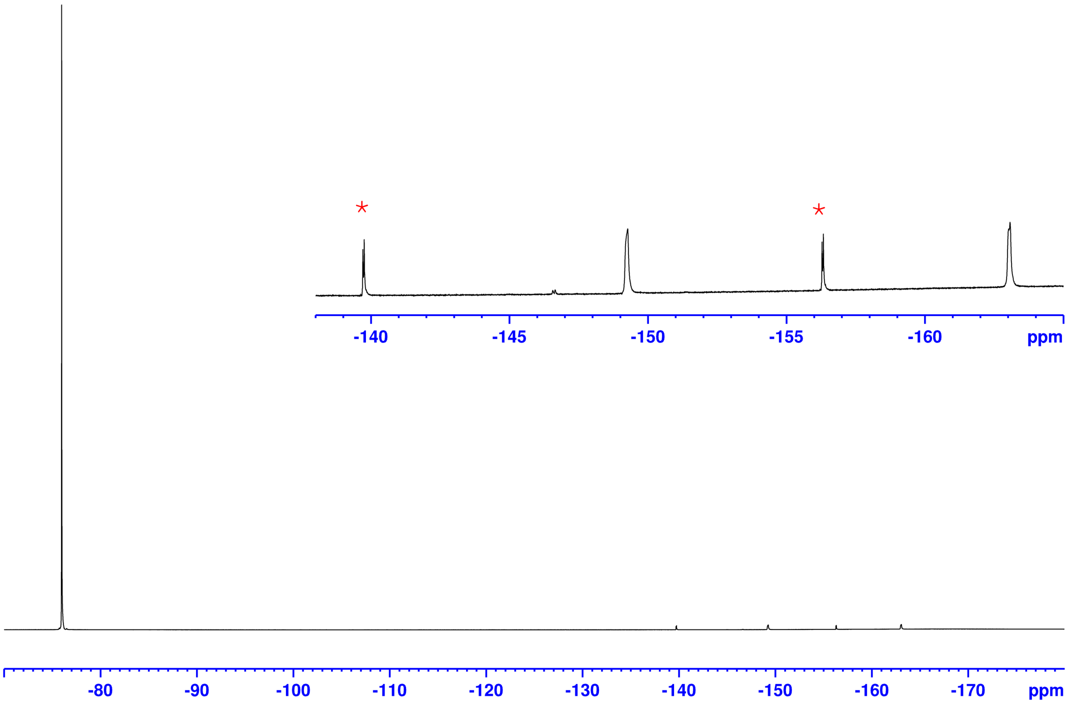

Figure S22: The $^{19}F\{^1H\}$ NMR (CD_2Cl_2, 183 K, 376 MHz) spectrum of **4a**. The inset is an enlargement of the bound and free $F_4C_6H_2$ resonances. Red asterisks denote free $F_4C_6H_2$ potentially signaling partial decomposition (ca. 25%).

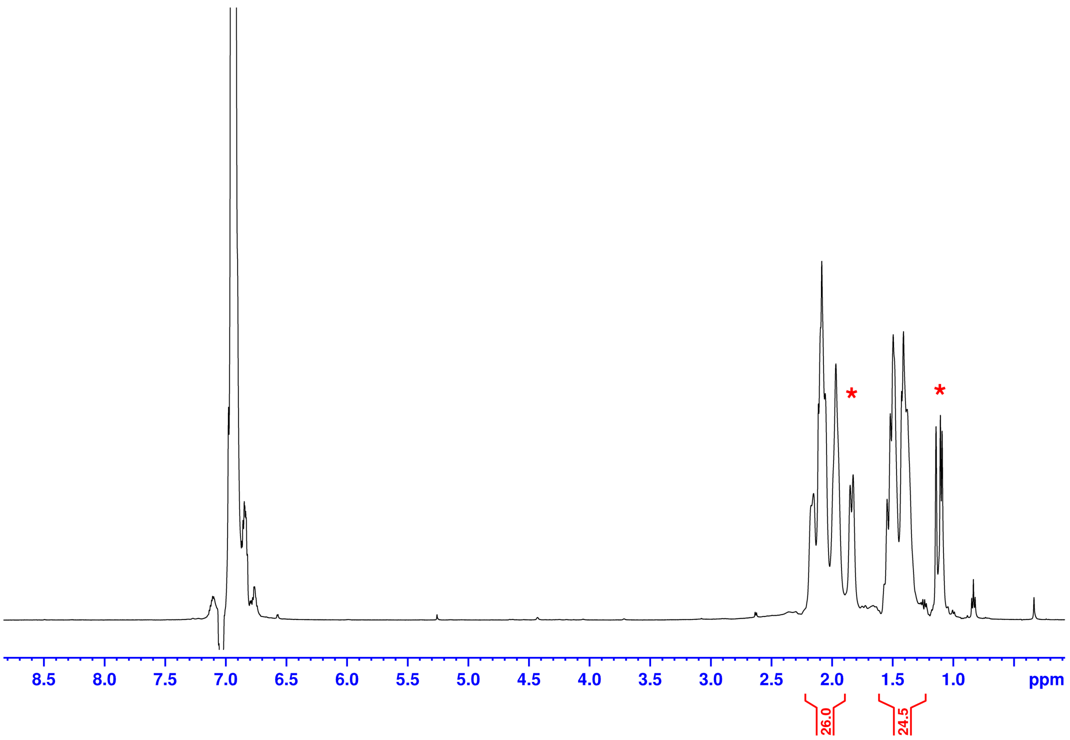

Figure S23: The ^{1}H NMR ($F_4C_6H_2$, 500 MHz) spectrum of **4b**. The red asterisks denote resonances for the NBA byproduct.

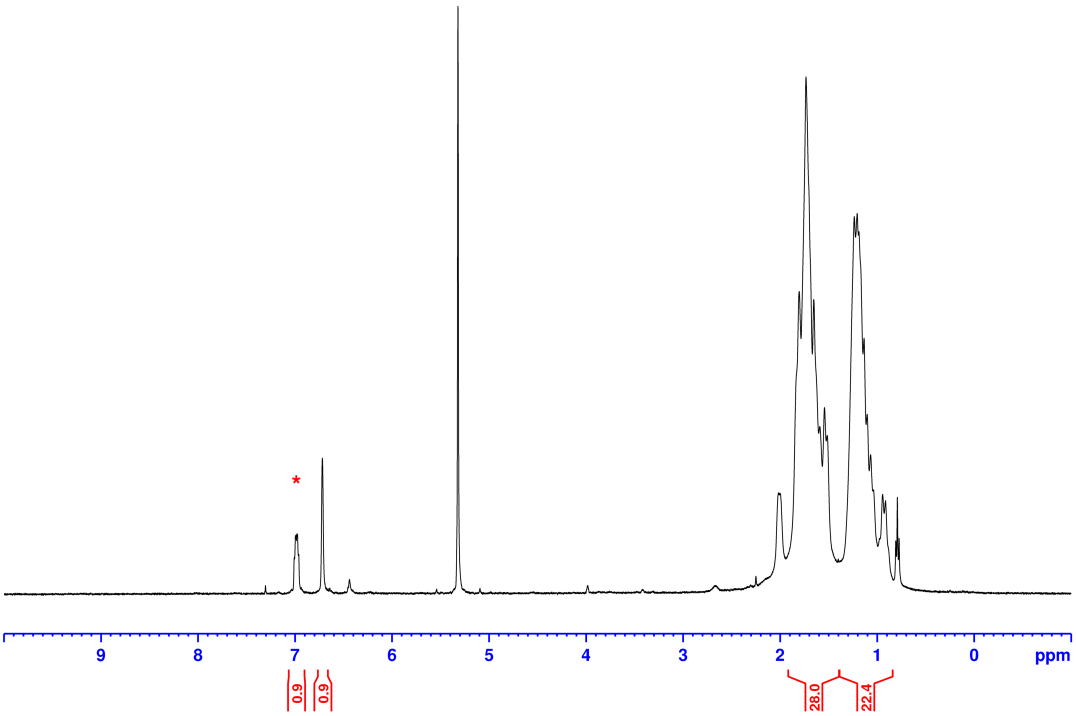

Figure S24: The ^{1}H NMR (CD_2Cl_2, 183 K, 400 MHz) spectrum of **4b**. The red asterisk denotes free $F_4C_6H_2$ potentially signaling partial decomposition (ca. 50%).

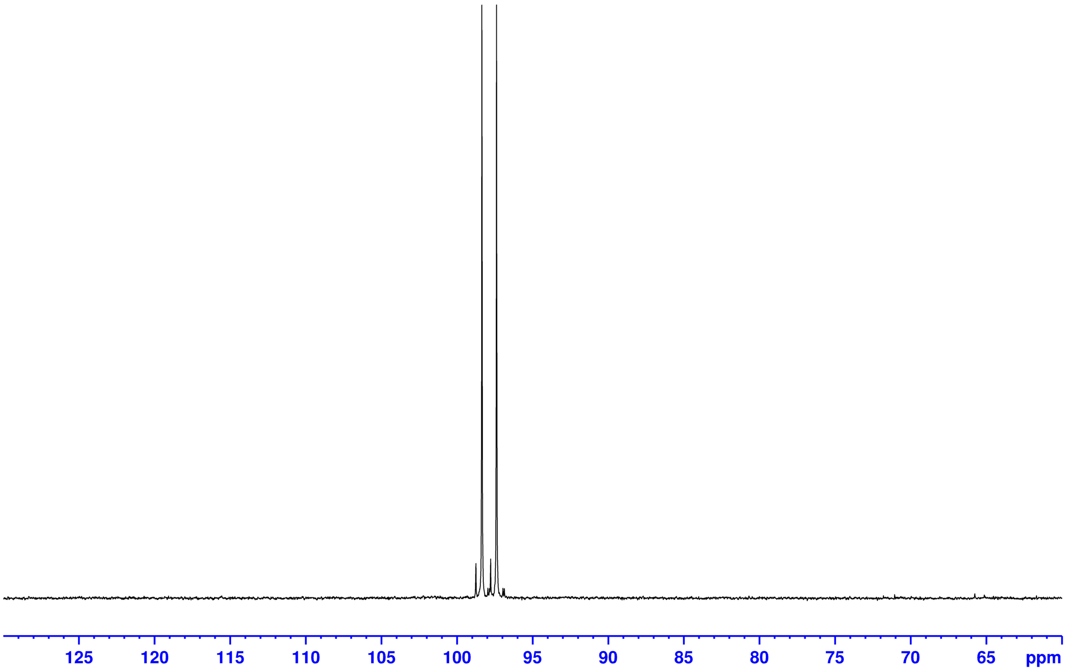

Figure S25: The $^{31}P\{^{1}H\}$ NMR ($F_4C_6H_2$, 202 MHz) spectrum of **4b**.

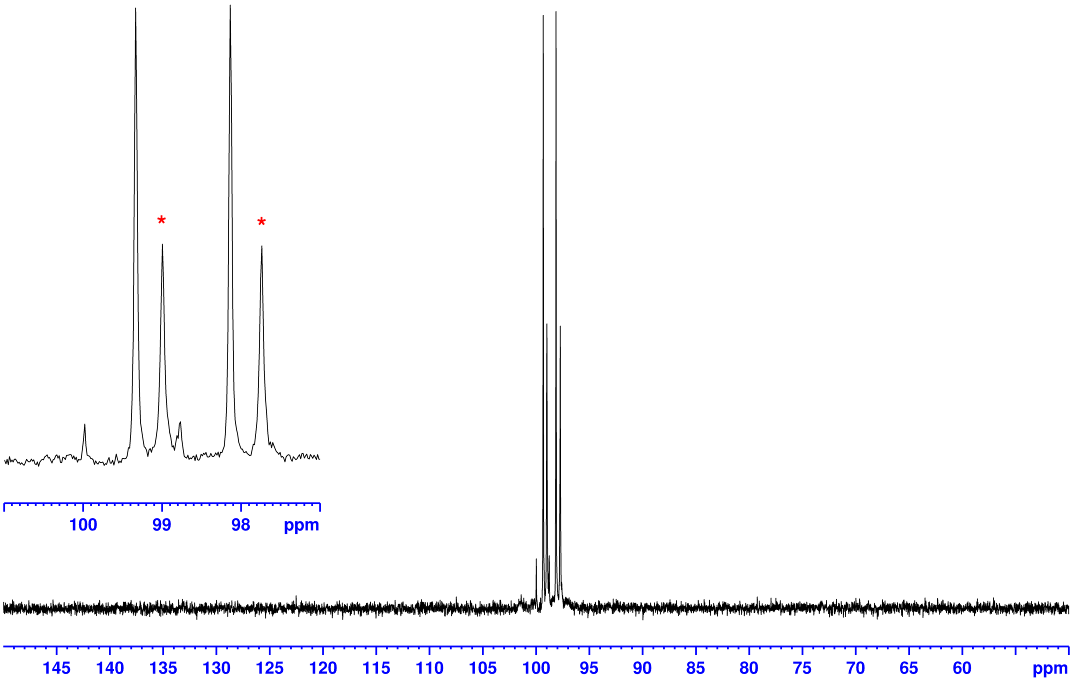

Figure S26: The $^{31}P\{^{1}H\}$ NMR (CD_2Cl_2, 183 K, 162 MHz) spectrum of **4b**. The inset is an enlargement of the 97-101 ppm region of the spectrum. The asterisks denote a doublet resonance (J_{RhP} 203 Hz) which we have previously tentatively assigned as a CD_2Cl_2 complex.[S2]

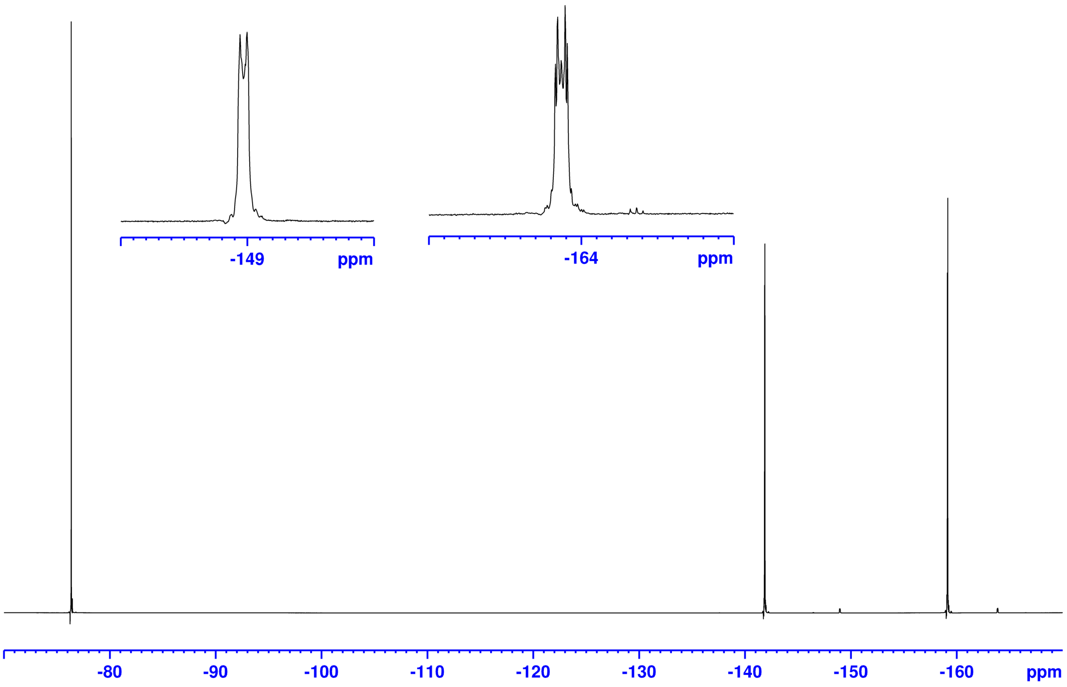

Figure S27: The $^{19}F\{^{1}H\}$ NMR ($F_4C_6H_2$, 470 MHz) spectrum of **4b**. The insets are enlargements of the bound $F_4C_6H_2$ resonances.

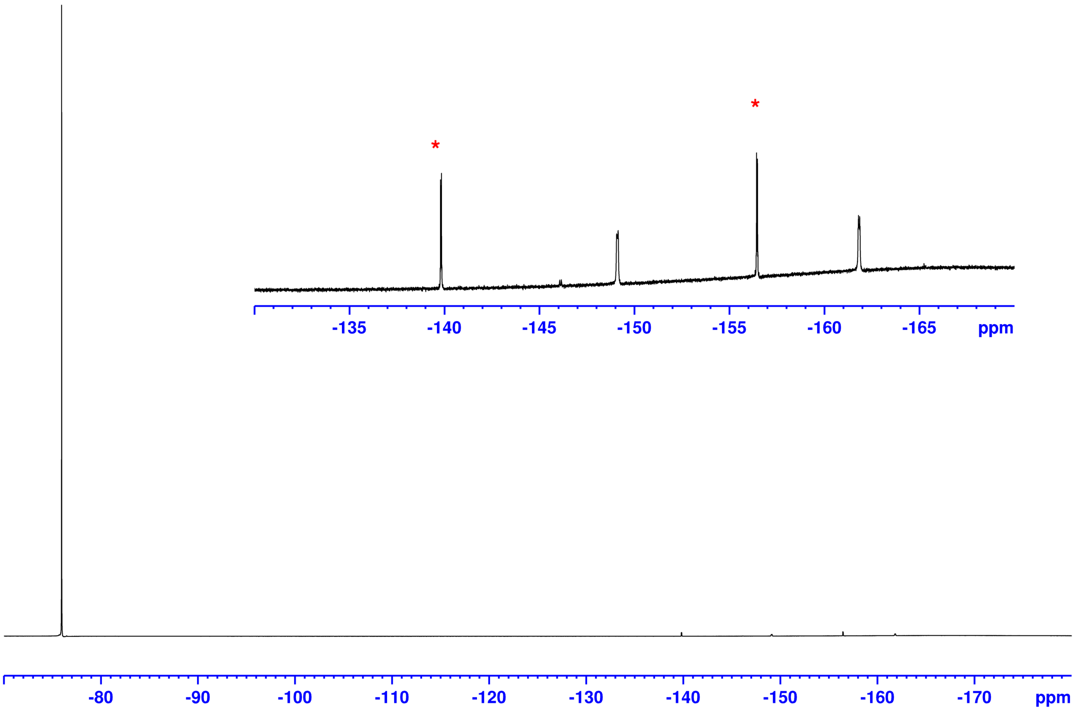

Figure S28: The $^{19}F\{^{1}H\}$ NMR (CD_2Cl_2, 183 K, 376 MHz) spectrum of **4b**. The inset is an enlargement of the bound and free $F_4C_6H_2$ resonances. Red asterisks denote free $F_4C_6H_2$ signaling partial decomposition (ca. 50%).

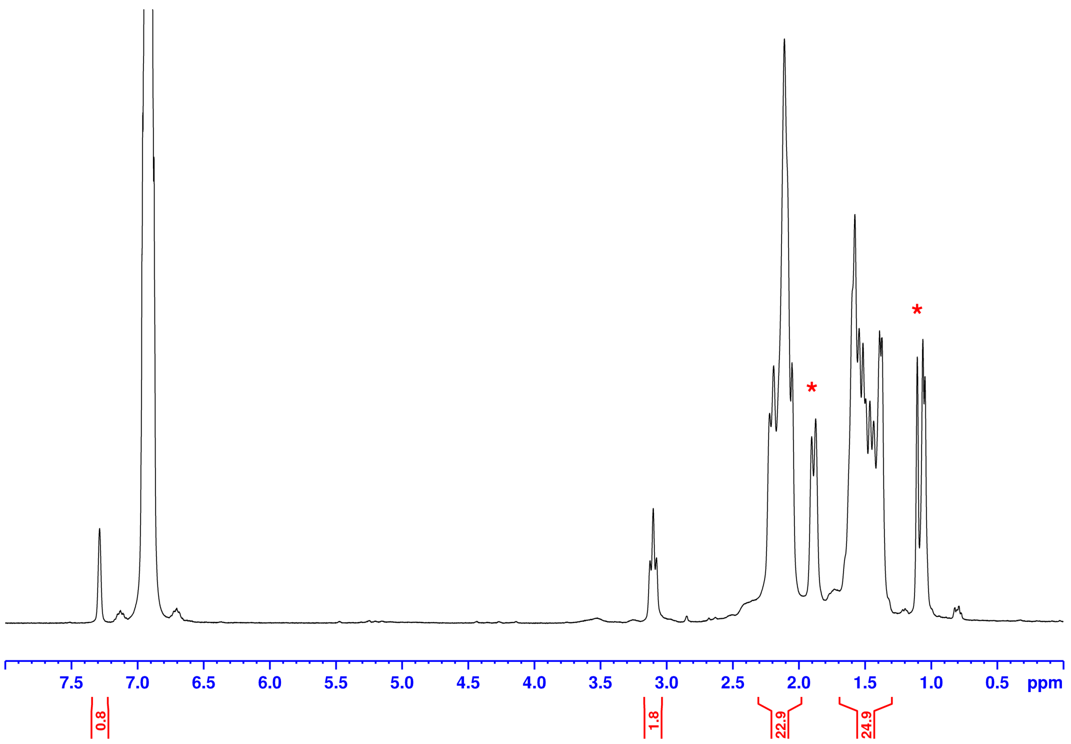

Figure S29: The ^{1}H NMR (F_5C_6H, 400 MHz) spectrum of **5a**. The red asterisks denote resonances for the NBA byproduct.

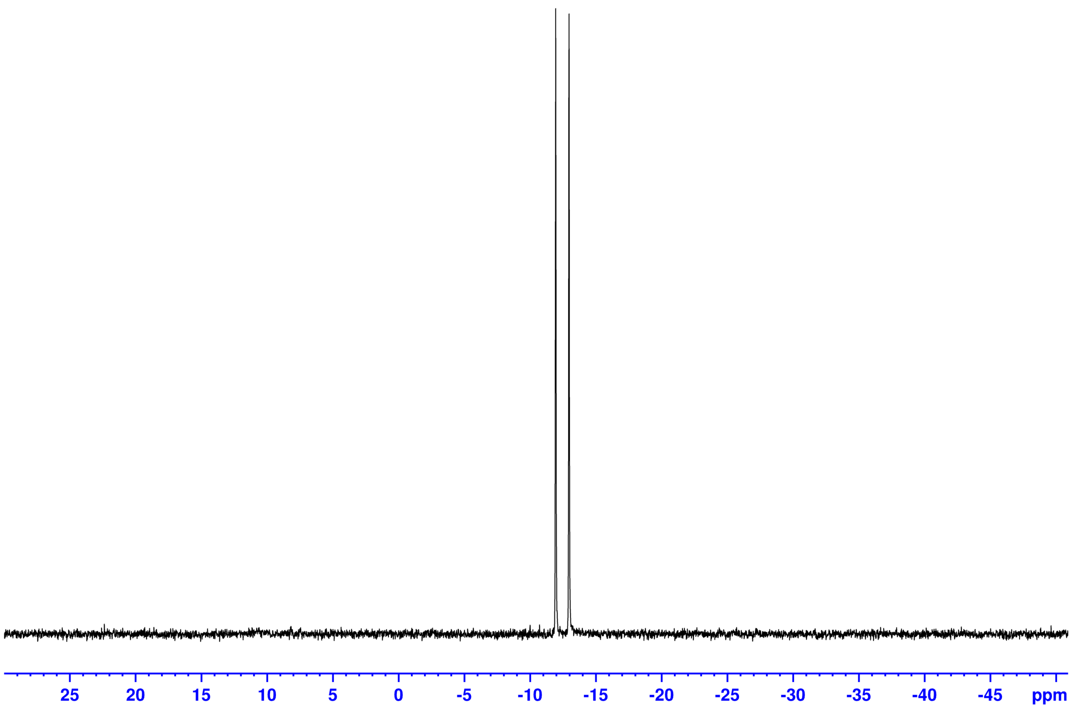

Figure S30: The $^{31}P\{^{1}H\}$ NMR (F_5C_6H, 162 MHz) spectrum of **5a**.

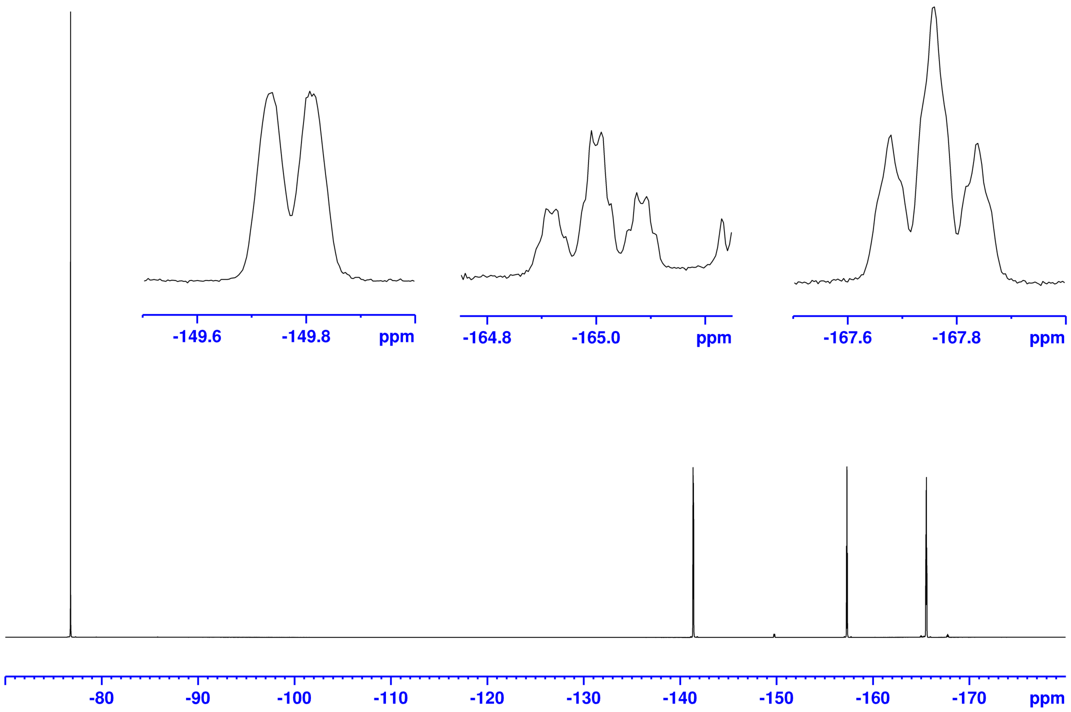

Figure S31: The $^{19}F\{^{1}H\}$ NMR (F_5C_6H, 376 MHz) spectrum of **5a**. The insets are enlargements of the bound F_5C_6H resonances.

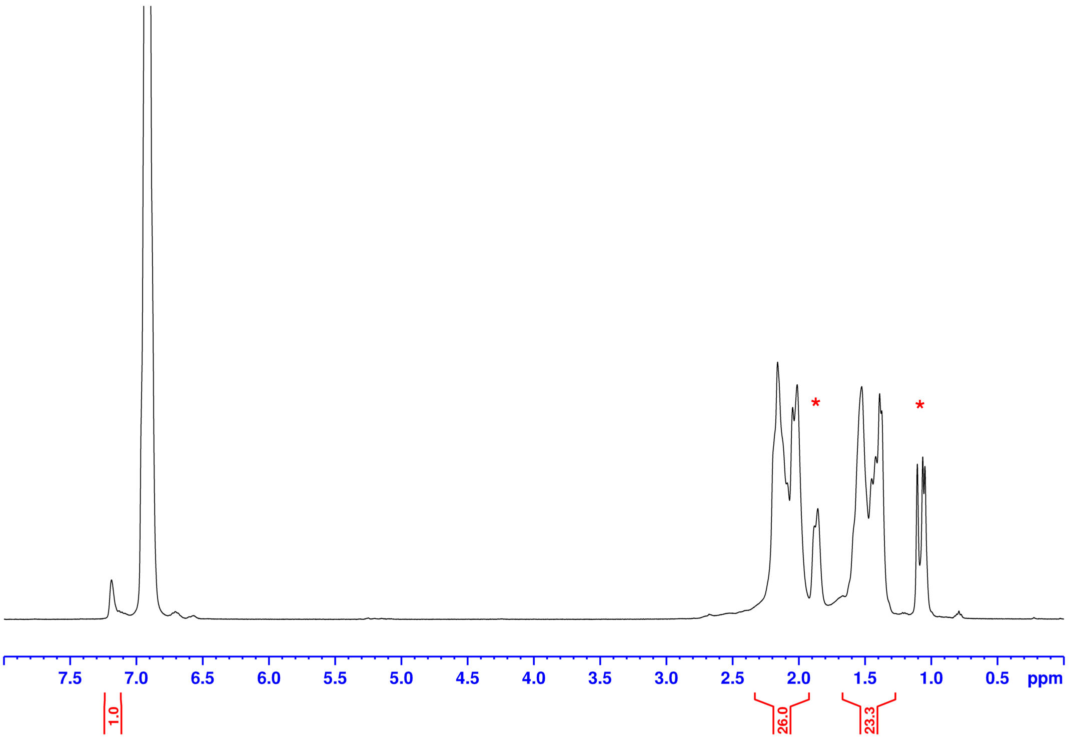

Figure S32: The ^{1}H NMR (F_5C_6H, 400 MHz) spectrum of **5b**. The red asterisks denote resonances for the NBA byproduct.

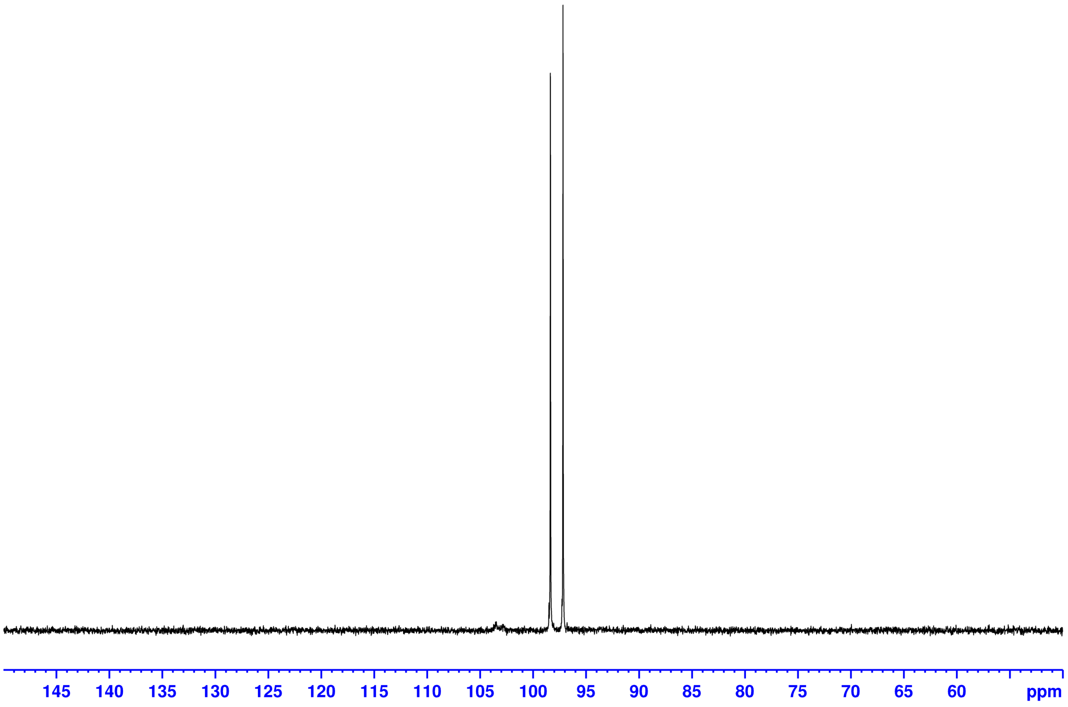

Figure S33: The $^{31}P\{^{1}H\}$ NMR (F_5C_6H, 162 MHz) spectrum of **5b**.

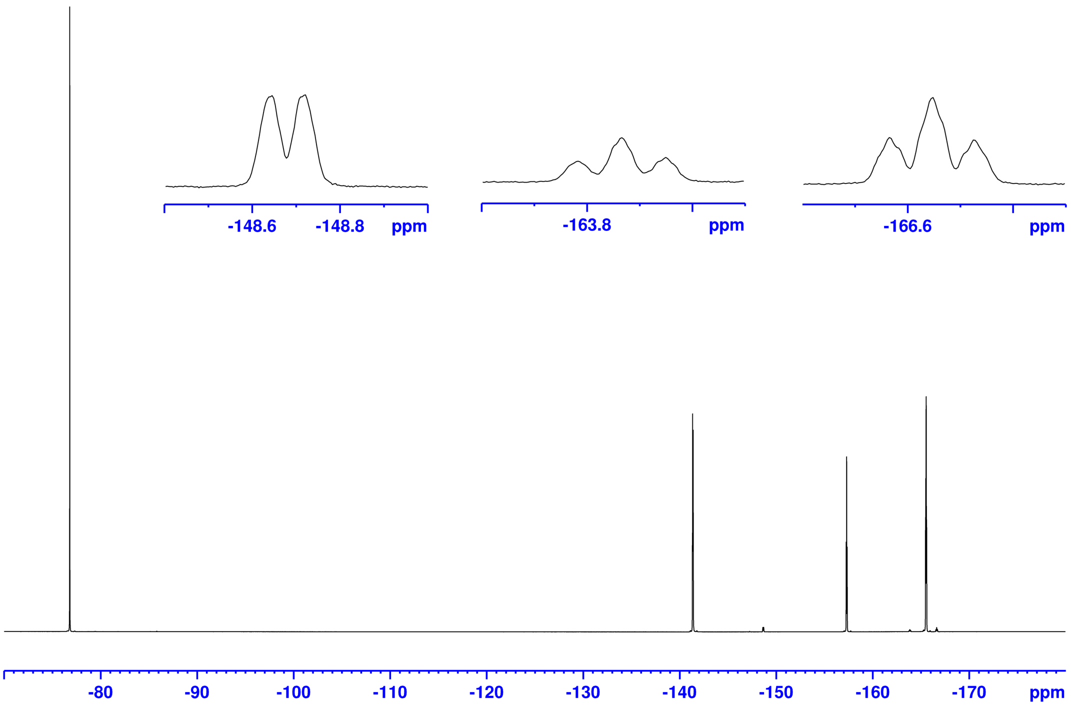

Figure S34: The $^{19}F\{^{1}H\}$ NMR (F_5C_6H, 376 MHz) spectrum of **5b**. The insets are enlargements of the bound F_5C_6H resonances.

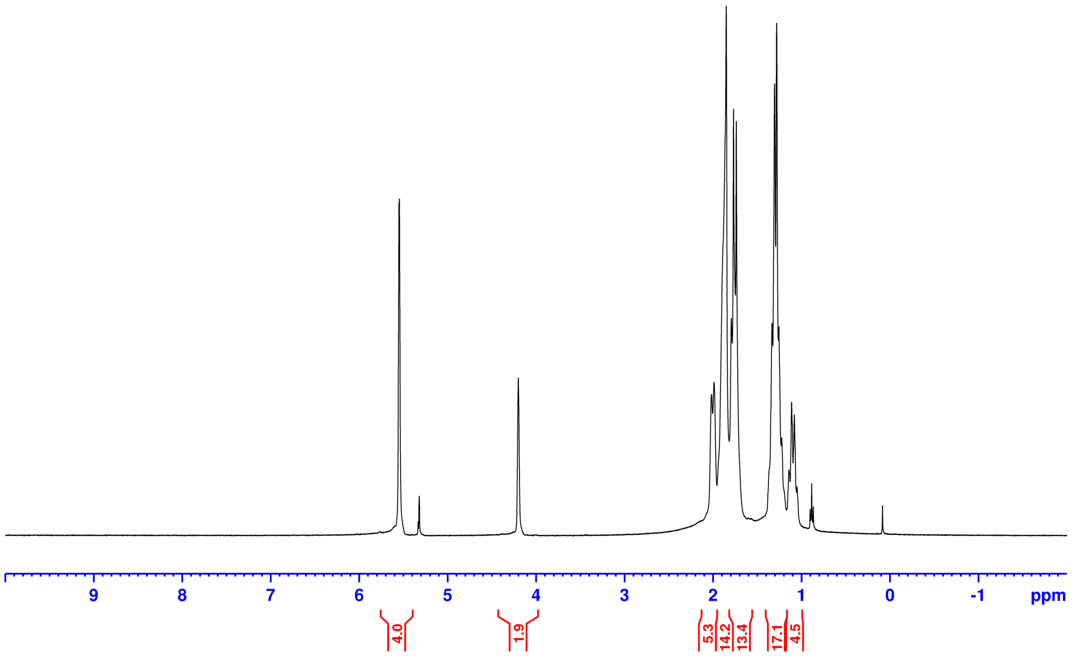

Figure S35: The ^{1}H NMR (CD_2Cl_2, 400 MHz) spectrum of **6a**.

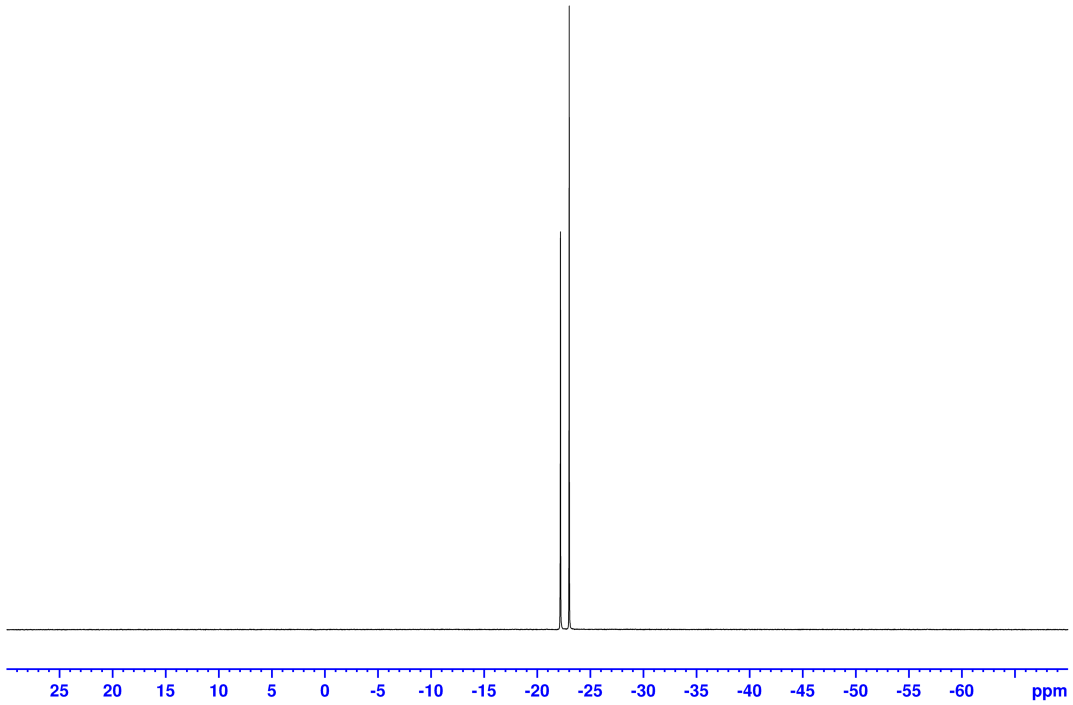

Figure S36: The ^{31}P{^{1}H} NMR (CD_2Cl_2, 162 MHz) spectrum of **6a**.

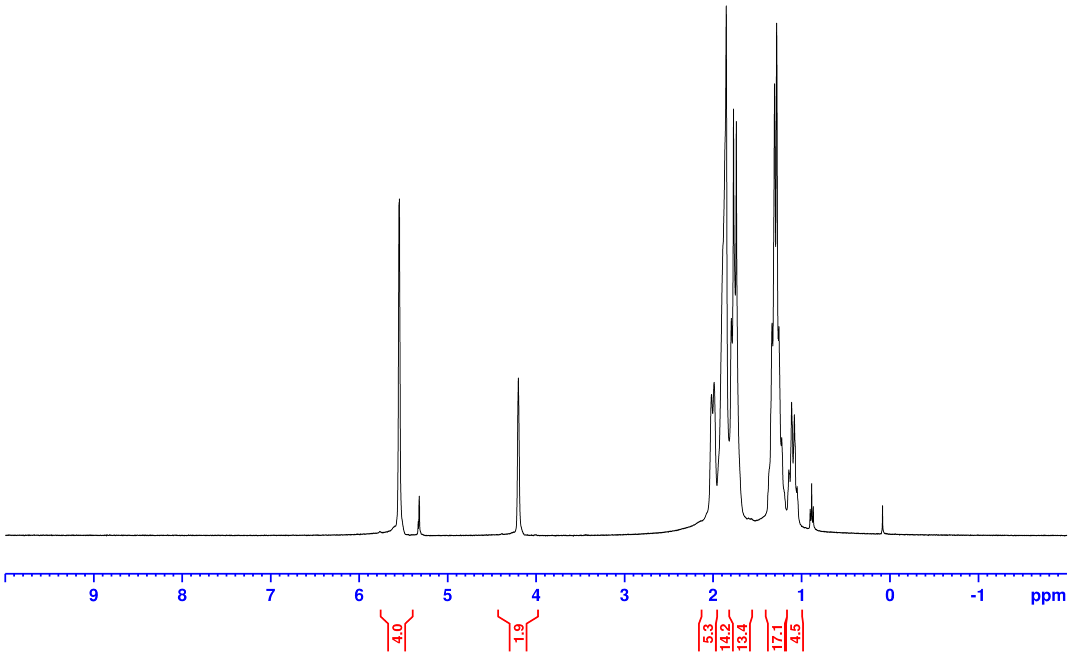

Figure S37: The ^{1}H NMR (CD_2Cl_2, 400 MHz) spectrum of **6b**.

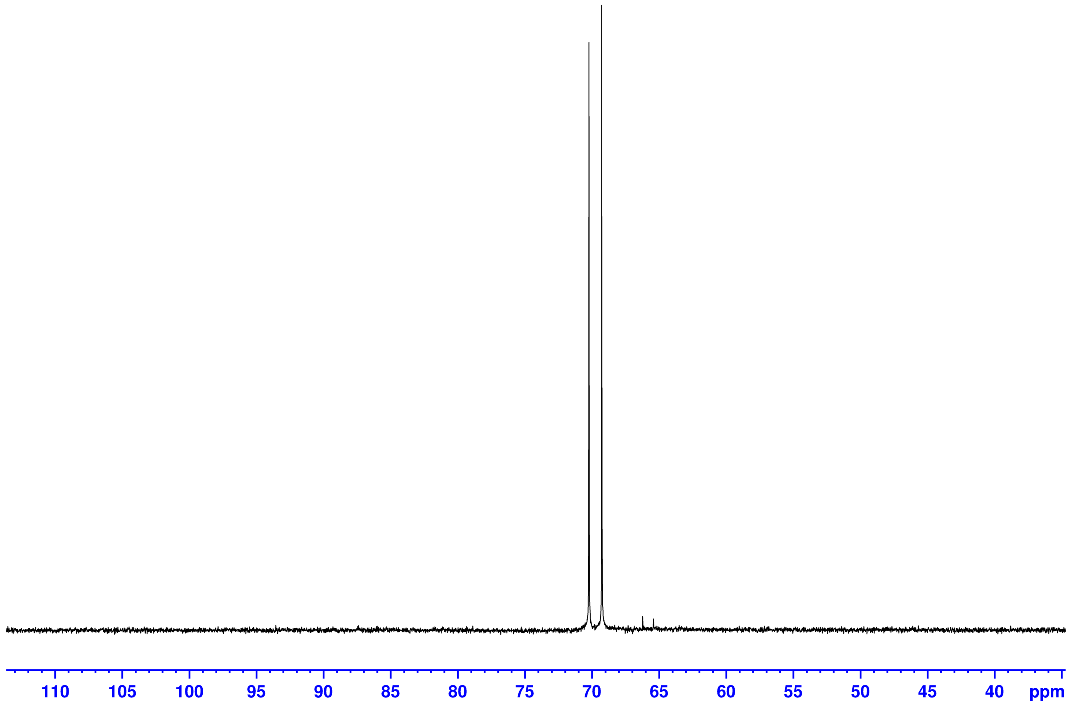

Figure S38: The $^{31}P\{^{1}H\}$ NMR (CD_2Cl_2, 162 MHz) spectrum of **6b**.

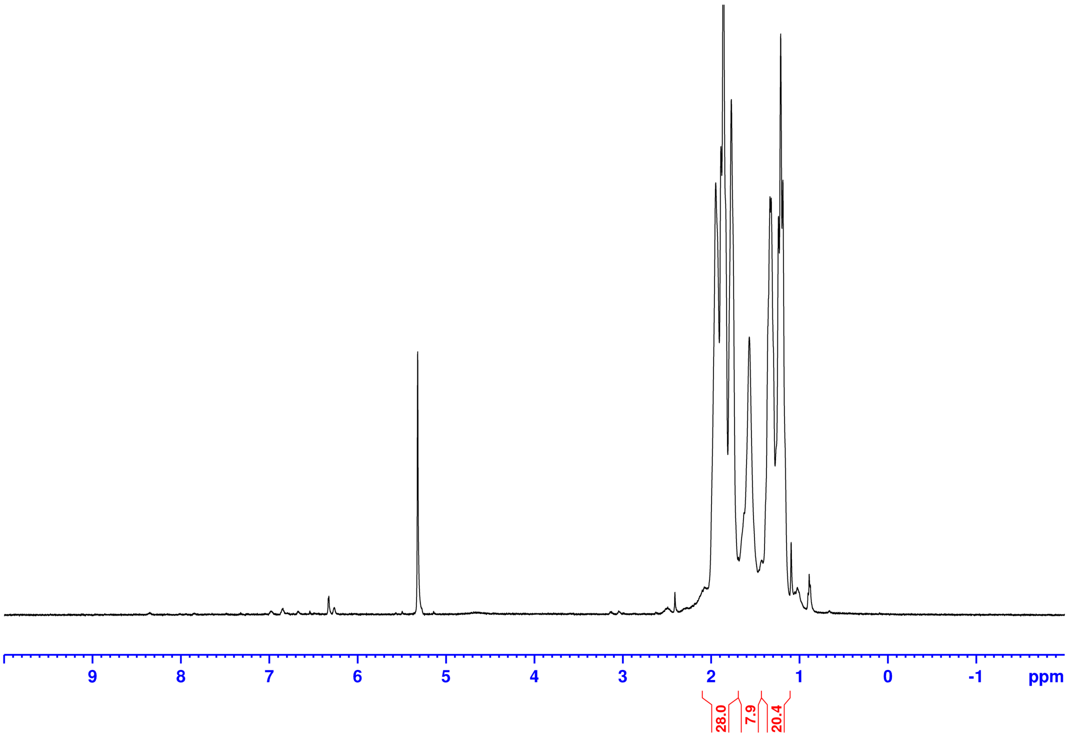

Figure S39: The ^{1}H NMR (CD_2Cl_2, 298 K, 500 MHz) spectrum of **7**.

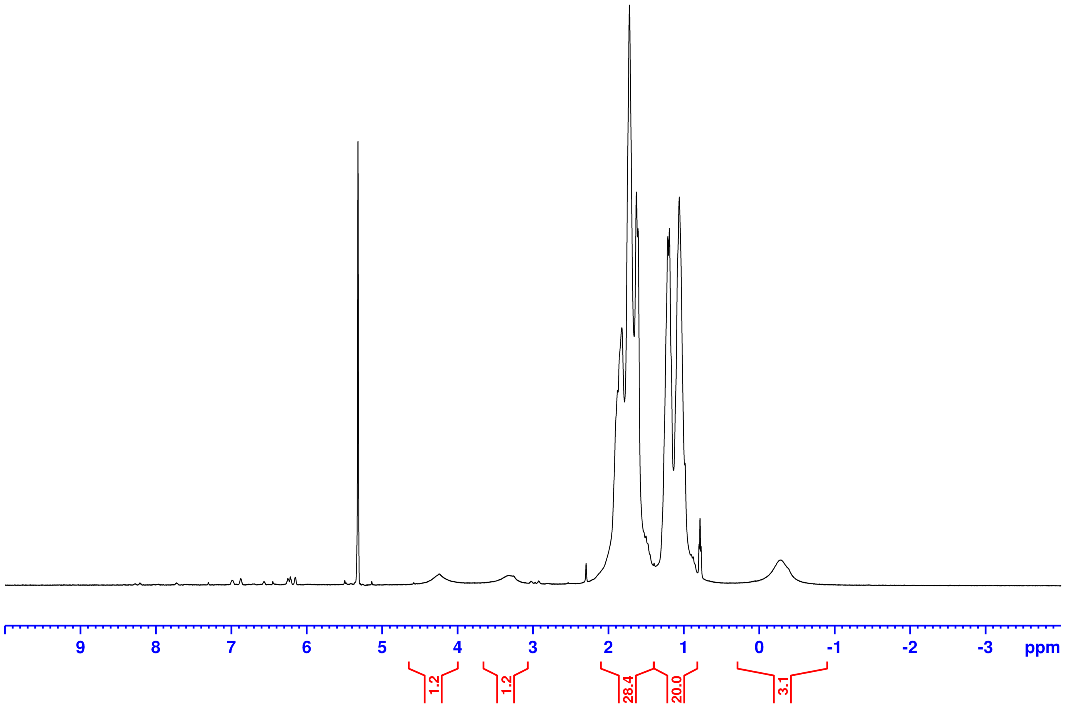

Figure S40: The ^{1}H NMR (CD_2Cl_2, 193 K, 500 MHz) spectrum of **7**.

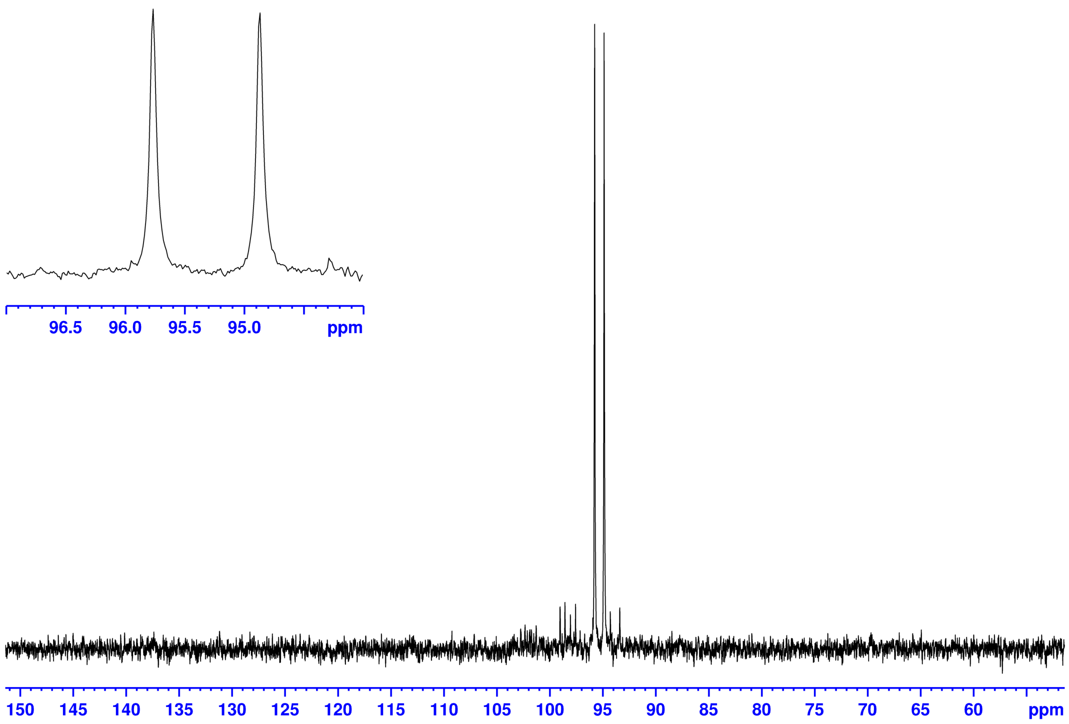

Figure S41: The $^{31}P\{^1H\}$ NMR (CD_2Cl_2, 298 K, 202 MHz) spectrum of **7**.

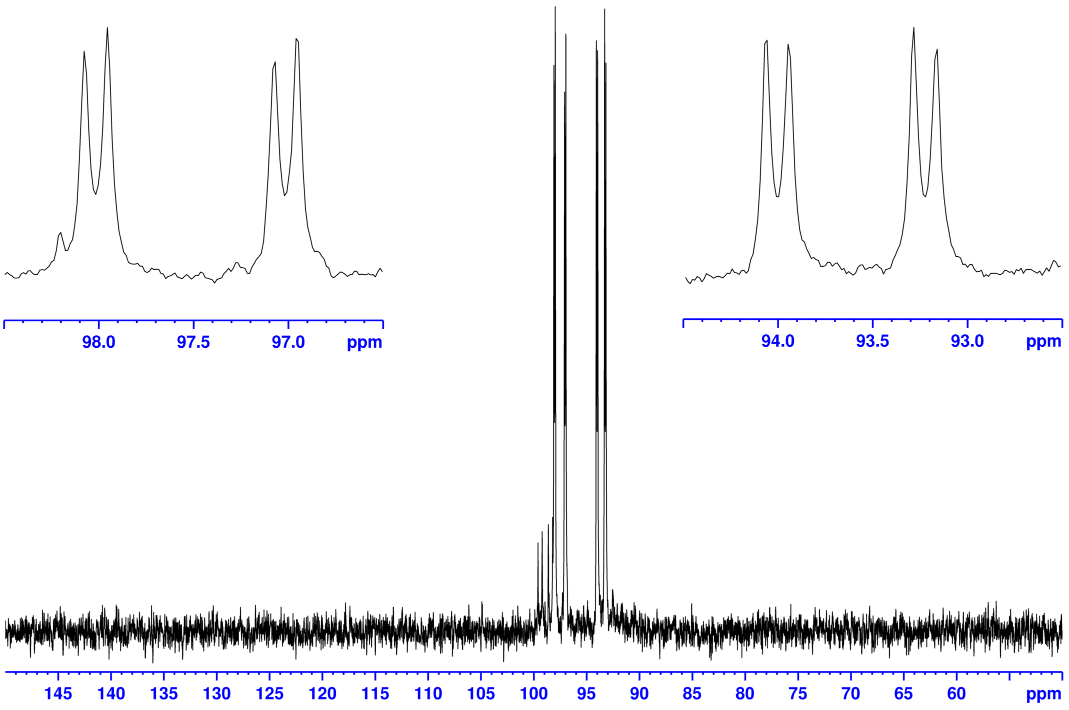

Figure S42: The $^{31}P\{^1H\}$ NMR (CD_2Cl_2, 193 K, 202 MHz) spectrum of **7**. The insets are enlargements of the resonances.

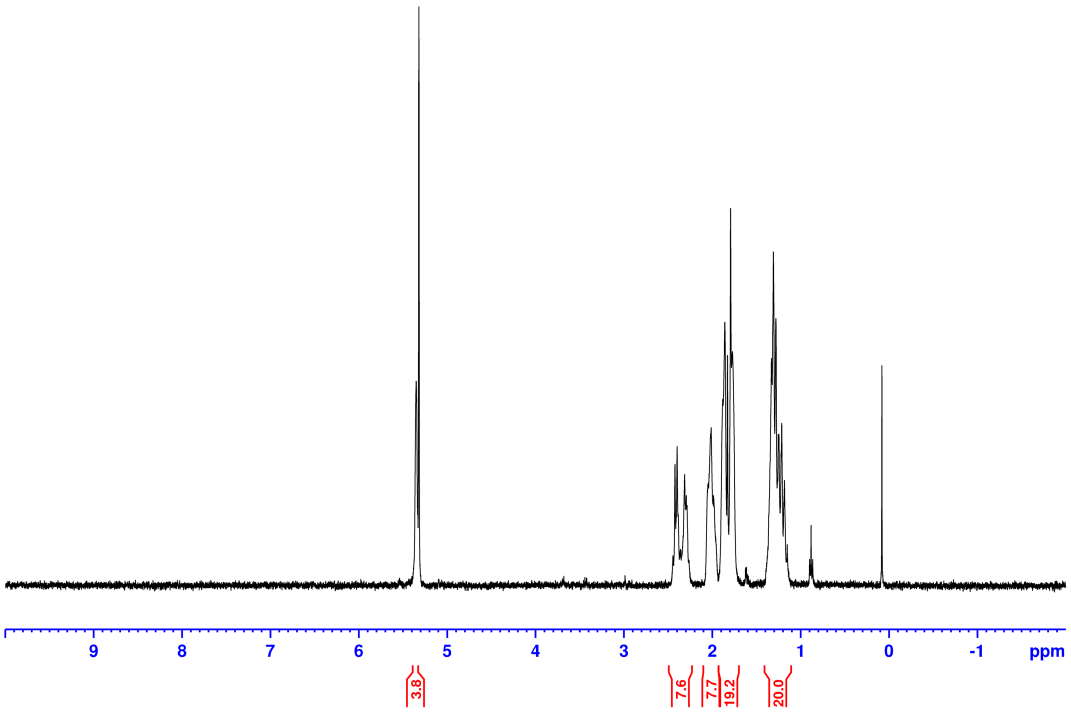

Figure S43: The ^{1}H NMR (CD_2Cl_2, 400 MHz) spectrum of **[Rh($Cy_2PCH_2CH_2PCy_2$)(COD)][Al{OC(CF_3)$_3$}$_4$]**.

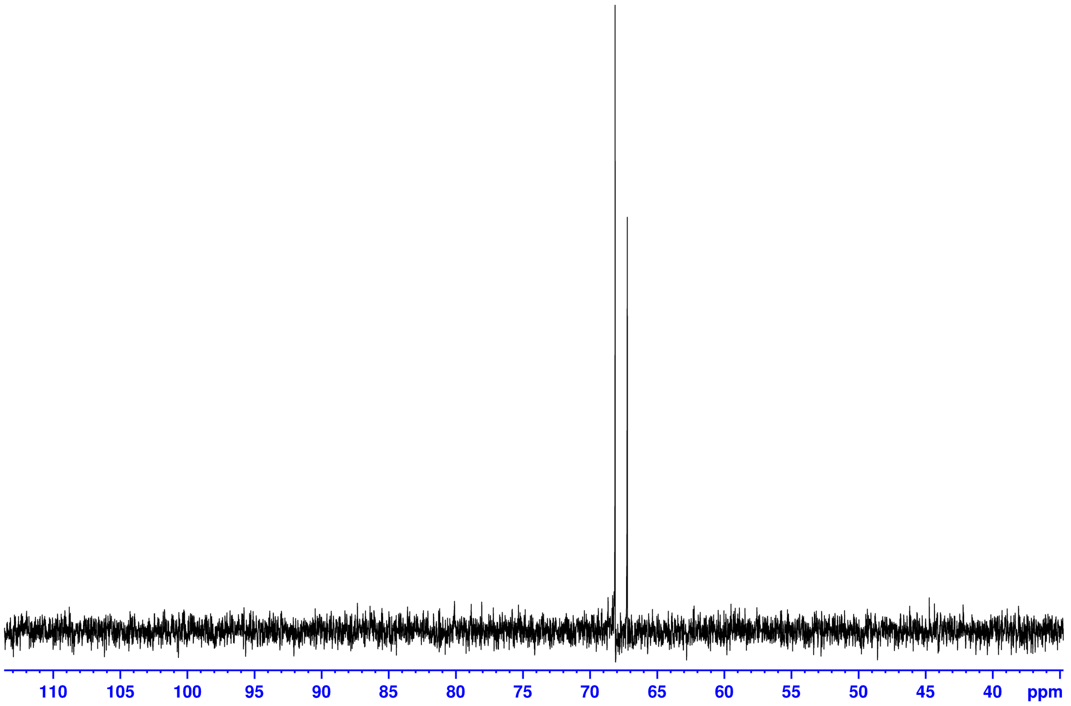

Figure S44: The ^{31}P{^{1}H} NMR (CD_2Cl_2, 162 MHz) spectrum of **[Rh($Cy_2PCH_2CH_2PCy_2$)(COD)][Al{OC(CF_3)$_3$}$_4$]**.

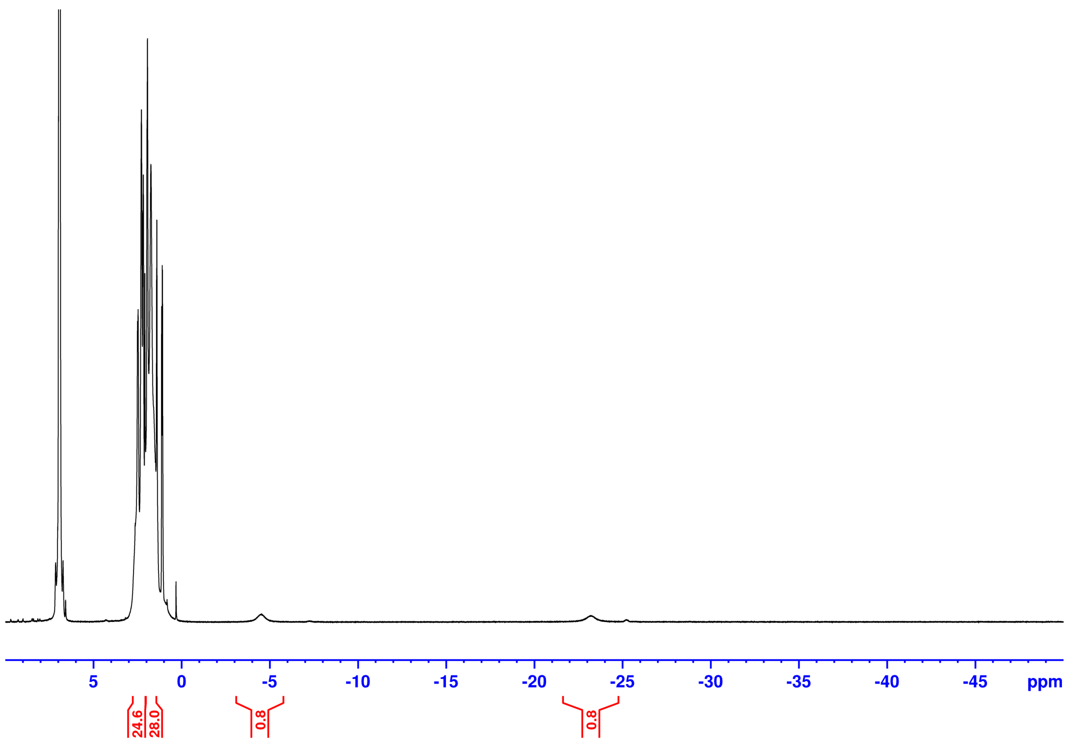

Figure S45: The ^{1}H NMR ($F_4C_6H_2$, 400 MHz) spectrum of the product obtained from the solid/gas reaction of **6b** with hydrogen.

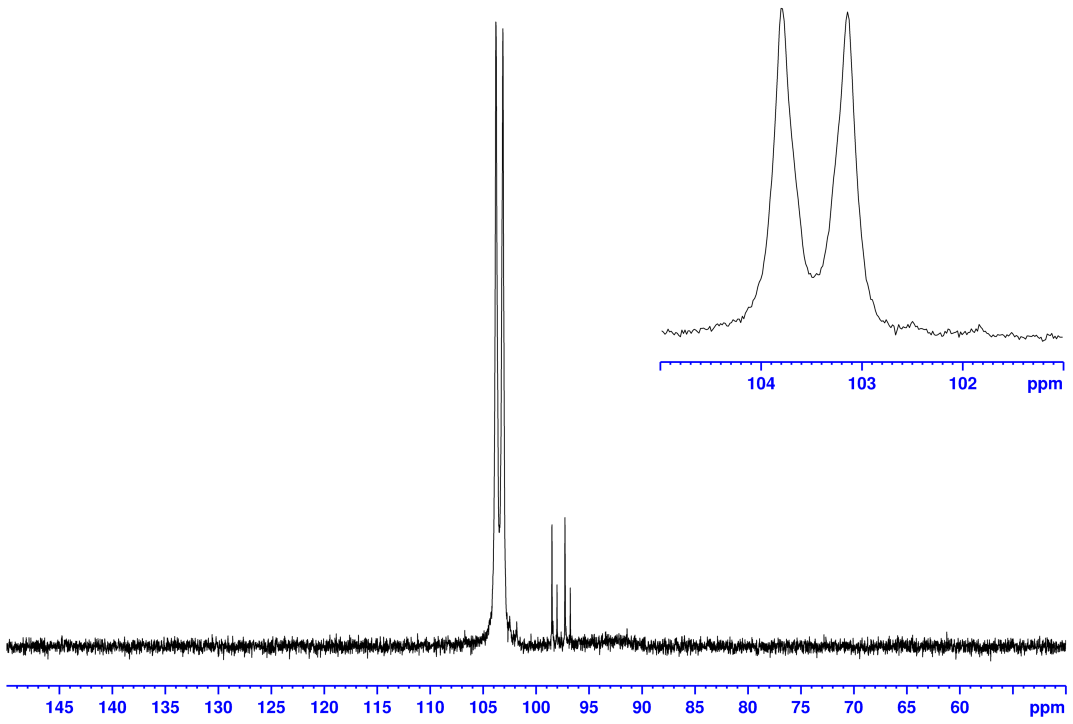

Figure S46: The $^{31}P\{^{1}H\}$ NMR ($F_4C_6H_2$, 162 MHz) spectrum of the product obtained from the solid/gas reaction of **6b** with hydrogen.

S.3. Crystallographic and refinement data

S.3.1. Crystal structure determinations

Single crystal X-ray diffraction data for all samples were collected as follows: a typical crystal was mounted on a MiTeGen micromounts using perfluoropolyether oil and cooled rapidly to 150 K in a stream of nitrogen gas using an Oxford Cryosystems unit.[S3] Data were collected with an Agilent SuperNova diffractometer (Cu K_{α} radiation, λ = 1.54180 Å). Raw frame data were reduced using CrysAlisPro.[S4] The structures were solved using SHELXT[S5] and refined using full-matrix least squares refinement on all F^2 data using the SHELXL-18[S6] using the interface OLEX2.[S7] All hydrogen atoms were placed in calculated positions (riding model). Rotational disorder of the PFB or the -$OC(CF_3)_3$ groups of the $Al\{OC(CF_3)_3\}_4$ anion were treated by modelling the atoms over multiple sites and restraining or constraining their geometries and displacement ellipsoids, with the aid of the FragmentDB module.[S8] Further comments regarding individual crystal structures can be found below. Crystallographic data have been deposited with the Cambridge Crystallographic Data Centre under CCDC 1958595-1958604. These data can be obtained free of charge from The Cambridge Crystallographic Data Centre via www.ccdc.cam.ac.uk/data_request/cif. Full bond length and bond angle data may be found in the CIFs.

S.3.2. Further comments on crystal structures

Platon Alerts (Poor Data / Parameter Ratio) are obtained for most structures particularly **5a**. In all instance, strong data and high completeness were obtained to a resolution of 0.80 Å. The anion and the PFB were found to be extensively disordered, necessitating a large number of parameters be employed in the refinement. Whilst refinement of disordered components employing isotropic displacement parameters afforded greatly improved the data / parameter ratio, the R_1 and wR_2 values deteriorated significantly. In the reported structures, disordered components were refined with anisotropic displacement parameters employing appropriate restrains. In all cases, despite the poor data / parameter ratios, the refinements were stable and converged.

S.3.3. Further crystal structures

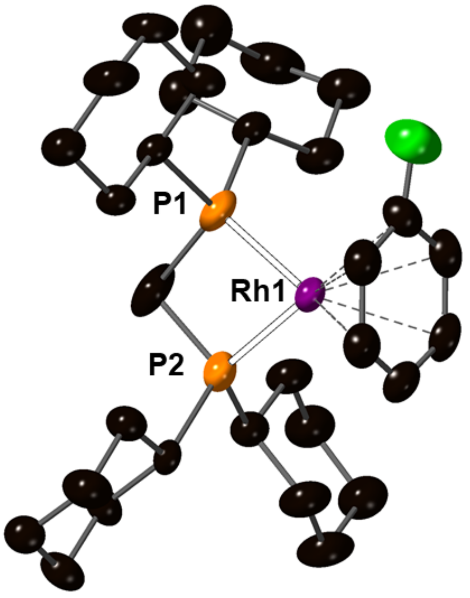

Figure S47: Molecular structure of **1a** with displacement ellipsoids at 50% probability. All lower occupancy disorder components and, hydrogen atoms have been omitted for clarity. Selected bond lengths (Å): Rh1-P1 2.2383(10), Rh1-P2 2.2324(10), Rh-C_{arene} range: 2.274(4)-2.340(5).

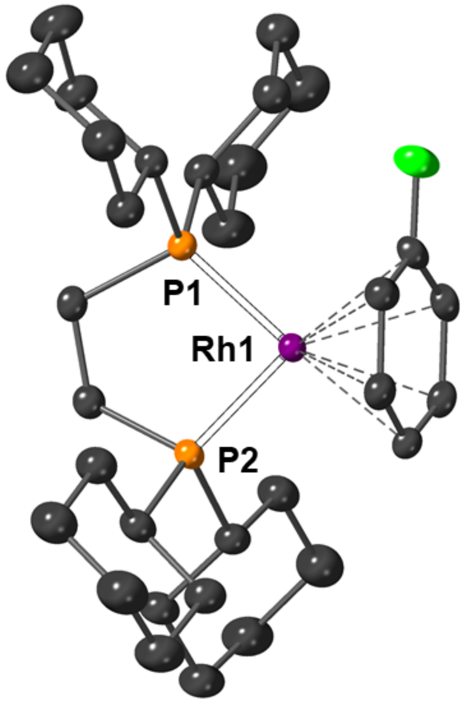

Figure S48: Molecular structure of **1b** with displacement ellipsoids at 50% probability. All lower occupancy disorder components and hydrogen atoms have been omitted for clarity. Selected bond lengths (Å): Rh1-P1 2.2405(7), Rh1-P2 2.2316(7), Rh-C_{arene} range: 2.280(5)-2.368(5).

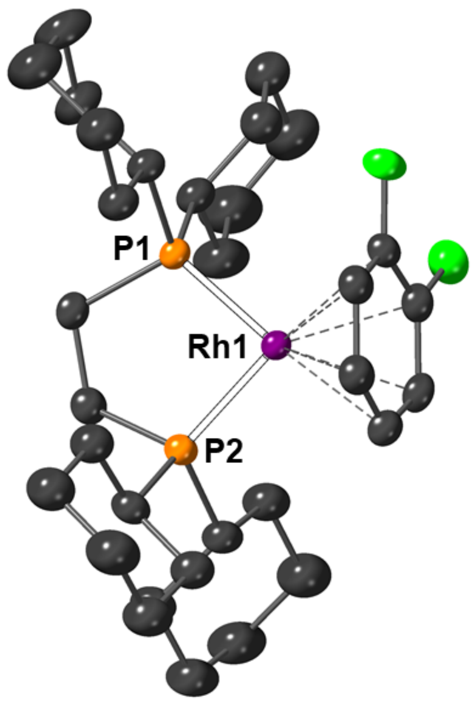

Figure S49: Molecular structure of **2b** with displacement ellipsoids at 50% probability. All lower occupancy disorder components and hydrogen atoms have been omitted for clarity. Selected bond lengths (Å): Selected bond lengths (Å): Rh1-P1 2.2394(8), Rh1-P2 2.2414(8), Rh-C_{arene} range: 2.279(3)-2.340(5).

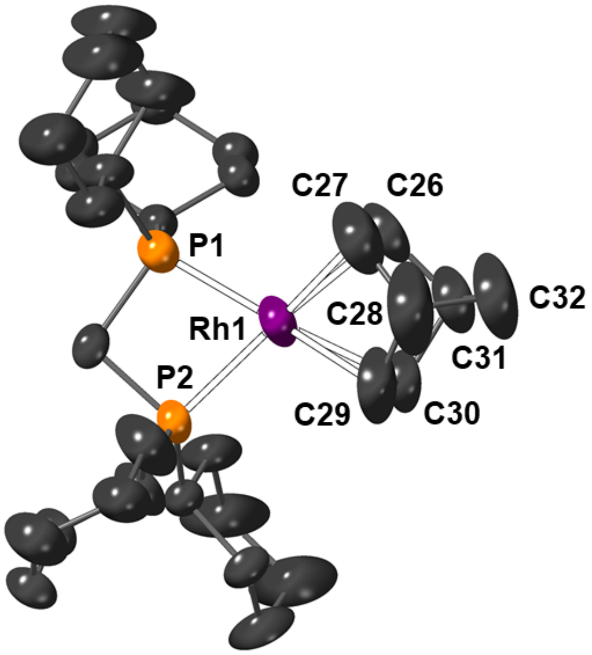

Figure S50: Molecular structure of **6a** with displacement ellipsoids at 50% probability displayed. All lower occupancy disorder components and hydrogen atoms and have been omitted for clarity. Selected bond lengths (Å): Rh1-P1 2.2934(17), Rh1-P2 2.3017(15), Rh-C26 2.210(7), Rh-C27 2.187(8), Rh-C29 2.201(7), Rh-C30 2.205(7).

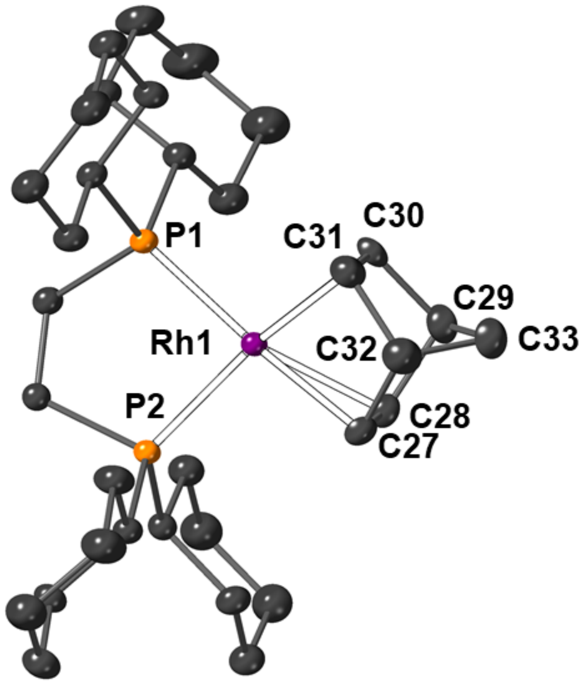

Figure S51: Molecular structure of **6b** with displacement ellipsoids at 50% probability displayed. All lower occupancy disorder components and hydrogen atoms and have been omitted for clarity. Selected bond lengths (Å): Rh1-P1 2.2894(5), Rh1-P2 2.2888(5), Rh-C27 2.212(2), Rh-C28 2.213(2), Rh-C30 2.216(2), Rh-C31 2.211(2).

Table S1: Selected crystallographic and refinement data.

	**1a**	**1b**	**2b**	**3b**	**4a**
Chemical formula	$C_{47}H_{51}AlF_{37}O_4P_2Rh$	$C_{48}H_{53}AlF_{37}O_4P_2Rh$	$C_{48}H_{52}AlF_{38}O_4P_2Rh$	$C_{48}H_{51}AlF_{39}O_4P_2Rh$	$C_{47}H_{48}AlF_{40}O_4P_2Rh$
Formula weight	1574.70	1588.73	1606.72	1624.71	1628.68
Crystal system	monoclinic	monoclinic	monoclinic	monoclinic	monoclinic
Space group	$P2_1/n$	$P2_1/c$	$P2_1/c$	$P2_1/c$	$P2_1/n$
a (Å)	18.6672(3)	16.9776(3)	16.7547(3)	16.7547(3)	18.8004(4)
b (Å)	15.9747(2)	17.5378(3)	17.6452(2)	17.6452(2)	16.0217(3)
c (Å)	21.0196(3)	20.2870(3)	20.6708(3)	20.6708(3)	21.1447(4)
α (deg)	90	90	90	90	90
β (deg)	108.632(2)	93.118(2)	94.7030(10)	94.7030(10)	108.902(2)
γ (deg)	90	90	90	90	90
V (Å^3)	5939.59(16)	6031.51(17)	6090.54(16)	6090.54(16)	6025.6(2)
Z	4	4	4	4	4
ρ(calcd) (g cm^{-3})	1.761	1.750	1.752	1.772	1.795
μ (mm^{-1})	4.457	4.395	4.385	4.418	4.492
F(000)	3144	3176	3209	3240	3240
Reflections collected	36041	51742	44426	379696	38501
Unique reflections	12313	12141	12329	12668	12511
Restraints / Parameters	1883/1178	2096/1283	4571/1241	2872/1229	2061/1334
R_{int}	0.0251	0.0330	0.0243	0.0533	0.0272
R_1 [$I > 2\sigma(I)$]	0.0622	0.0467	0.0525	0.0535	0.0611
wR_2 [all data]	0.17602	0.1281	0.1444	0.1489	0.1782
GooF	1.052	1.038	1.033	1.038	1.033
Residual electron density (e Å^{-3})	1.50/-1.13	1.32/-0.74	1.27/-0.77	1.26/-0.97	1.33/-0.90
CCDC no.	1958595	1958596	1958597	1958598	1958599

Table S2: Selected crystallographic and refinement data.

	**4b**	**5a**	**6a**	**6b**	**7**
Chemical formula	$C_{48}H_{50}AlF_{40}O_4P_2Rh$	$C_{47}H_{47}AlF_{41}O_4P_2Rh$	$C_{48}H_{54}AlF_{36}O_4P_2Rh$	$C_{49}H_{56}AlF_{36}O_4P_2Rh$	$C_{46}H_{56}AlF_{36}O_4P_2Rh$
Formula weight	1642.71	1646.67	1570.74	1584.76	1548.73
Crystal system	monoclinic	orthorhombic	monoclinic	orthorhombic	triclinic
Space group	$P2_1/c$	$P2_12_12_1$	$P2_1/n$	Pbca	P-1
a (Å)	16.7301(2)	15.7498(2)	18.4581(3)	19.25580(16)	12.2199(4)
b (Å)	17.6506(2)	18.7469(2)	16.2468(2)	20.42700(17)	15.3044(5)
c (Å)	20.7432(3)	20.5842(3)	21.2808(4)	30.7197(3)	16.5407(6)
α (deg)	90	90	90	90	88.969(3)
β (deg)	95.2600(10)	90	107.741(2)	90	82.075(3)
γ (deg)	90	90	90	90	76.456(3)
V (Å^3)	6099.60(13)	6077.69(13)	6078.30(18)	12083.23(17)	2978.34(18)
Z	4	4	4	8	2
ρ(calcd) (g cm^{-3})	1.789	1.800	1.716	1.742	1.727
μ (mm^{-1})	4.444	4.487	4.328	4.361	4.405
F(000)	3272	3272	3144	6352	1552
Reflections collected	38938	52644	24609	96794	31382
Unique reflections	12611	12525	11508	12269	12271
Restraints / Parameters	1320/1119	3308/1302	2623/1392	884/965	1246/1067
R_{int}	0.0458	0.0394	0.0263	0.0261	0.0327
R_1 [$I > 2\sigma(I)$]	0.0657	0.0466	0.0893	0.0368	0.0397
wR_2 [all data]	0.1940	0.1313	0.2456	0.0992	0.1048
GooF	1.042	1.040	1.054	1.018	1.036
Residual electron density (e Å^{-3})	1.56/-1.02	0.92/-0.81	1.82/-1.90	1.63/-0.50	1.58/-0.48
CCDC no.	1958560	1958561	1958562	1958563	1958564

S.4. References

[S1] [Rh($Cy_2PCH_2CH_2PCy_2$)(COD)][Al{OC(CF_3)$_3$}$_4$] was prepared by a similar method to that used to prepare [Rh($Cy_2PCH_2CH_2PCy_2$)(COD)][$BAr^F{}_4$]. See Pike, S. D.; Chadwick, F. M.; Rees, N. H.; Scott, M. P.; Weller, A. S.; Krämer, T.; Macgregor, S. A., *J. Am. Chem. Soc.* **2015**, *137*, 820-833.

[S2] Chadwick, F. M.; Rees, N. H.; Weller, A. S.; Krämer, T.; Iannuzzi, M.; Macgregor, S. A., *Angew. Chem. Int. Ed.* **2016**, *55*, 3677-3681.

[S3] Cosier, J.; Glazer, A. M., *J. Appl. Cryst.* **1986**, *19*, 105-107.

[S4] Oxford Diffraction Ltd., Oxford Diffraction Ltd., 2011.

[S5] Sheldrick, G. M., *Acta Cryst.* **2015**, *A71*, 3-8.

[S6] Sheldrick, G. M., *Acta Cryst.* **2015**, *C71*, 3-8.

[S7] Dolomanov, O. V.; Bourhis, L. J.; Gildea, R. J.; Howard, J. A. K.; Puschmann, H., *J. Appl. Cryst.* **2009**, *42*, 339-341.

[S8] (a) Kratzert, D.; Holstein, J. J.; Krossing, I., *J. Appl. Cryst.* **2015**, *48*, 933-938; (b) Kratzert, D.; Krossing, I., *J. Appl. Cryst.* **2018**, *51*, 928-934.
